# Klf4-Sirt3/Pparα-Lcad pathway contributes to high phosphate-induced lipid degradation

**DOI:** 10.1186/s12964-022-01008-w

**Published:** 2023-01-09

**Authors:** Angen Yu, Yichuang Xu, Christer Hogstrand, Tao Zhao, Xiao-Ying Tan, Xiaolei Wei, Yu-Feng Song, Zhi Luo

**Affiliations:** 1 Hubei Hongshan Laboratory, Fishery College, Huazhong Agricultural University, Wuhan, 430070 China; 2 Laboratory for Marine Fisheries Science and Food Production Processes, Qingdao National Laboratory for Marine Science and Technology, Qingdao, 266237 China; 3 Diabetes and Nutritional Sciences Division, School of Medicine, King’s College London, Franklin-Wilkins Building, 150 Stamford Street, London, SE1 9NH UK

**Keywords:** Phosphorus, Klf4-Sirt3/Pparα-Lcad pathway, Fatty acid β-oxidation, Lipolysis, Molecular Nutrition

## Abstract

**Background:**

Phosphorus commonly reduces lipid deposition in the vertebrates. However, the underlying mechanisms involved in the process remain unclear.

**Methods:**

Yellow catfish were given three experimental diets with dietary phosphate levels of 3.22, 6.47 and 7.99 g Pi kg^− 1^, respectively, for 8 weeks. The contents of triglyceride, non-esterified free fatty acids, adenosine triphosphate, nicotinamide adenine dinucleotide, nicotinamide adenine dinucleotide, enzymatic activities, mRNA and protein expression were determined in the intestinal tissues. Hematoxylin and eosin, Oil Red O staining, and transmission electron microscope were performed for intestinal tissues. Primary intestinal epithelial cells were isolated from yellow catfish intestine. Western blot analysis, Immunoprecipitation assays, Immunofluorescence staining, and RNA extraction and quantitative real-time PCR were decided. Luciferase reporter assays and electrophoretic mobility shift assay were used to evaluate the function of Sirt3, PPARα and Lcad promoters.

**Results:**

High dietary phosphate intake activated intestinal phosphate absorption and excretion, and reduced lipid deposition through increasing lipolysis in the intestine. Moreover, phosphate incubation increased the mRNA and protein expression of *krüppel like factor 4* (*klf4)*, *silent mating-type information regulation 2 homolog 3* (*sirt3)*, *peroxisome proliferator activated receptor alpha (pparα)* and *long chain acyl-CoA dehydrogenase (lcad)* in the intestinal epithelial cells (IECs), and *klf4* knockdown attenuated the phosphate-induced increase of protein levels of Sirt3, Pparα and Lcad. Further investigation found that Klf4 overexpression increased the activity of *sirt3* and *pparα* promoters, which in turn reduced the acetylation and protein level of Lcad.

**Conclusion:**

Dietary Pi excess induced lipid degradation by the activation of the Klf4-Sirt3/Pparα-Lcad pathway in the intestine and primary IECs.

**Graphical Abstract:**

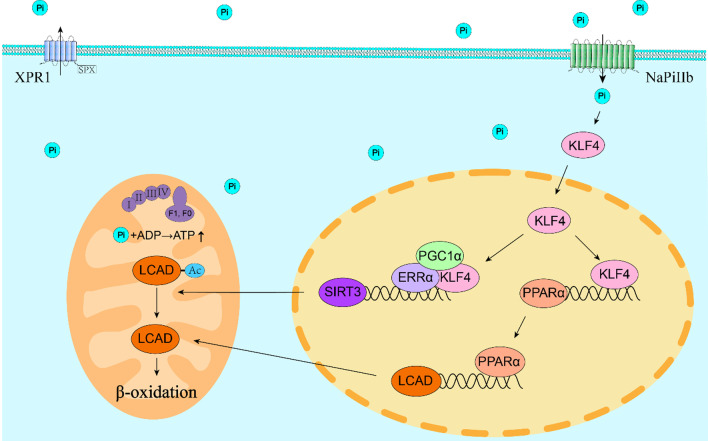

**Video Abstract**

**Supplementary Information:**

The online version contains supplementary material available at 10.1186/s12964-022-01008-w.

## Introduction

Phosphorus is a predominant mineral element within the body of animals and plays many vital roles in diverse biological processes, including skeletal formation, the maintenance of cell membrane integrity, ATP synthesis and lipid metabolism [1]. The intestine is the principal organ for phosphate absorption. In mammals, the type II sodium-dependent phosphate cotransporters, including SLC34A1, SLC34A2 and SLC34A3, and the type III sodium-dependent phosphate cotransporters, including PiT-1 and PiT-2, are the main phosphate transporters who transport extracellular phosphate [1, 2]. The mitochondrial phosphate carrier protein (PIC) and uncoupling protein 2 (UCP2) transport inorganic phosphate into the mitochondrial matrix and contribute to ATP synthesis under high phosphate incubation [3, 4]. The xenotropic and polytropic retrovirus receptor 1 (XPR1) ensures inorganic phosphate efflux under high phosphate incubation [5]. At present, although studies explored influences of dietary phosphate on the expression of genes and proteins relevant with phosphorus absorption, the underlying mechanism for intestinal Pi regulation in responses to dietary phosphate addition remains unclear.

On the other hand, inorganic phosphorus (Pi) is a key component such as adenosine tri-phosphorus (ATP) and plays important roles in maintenance of mitochondrial homeostasis [6, 7]. Mitochondria act as the cell’s power plant and produce ATP via oxidative phosphorylation (OXPHOS). Notably, mitochondria are also important organelles for fatty acid β-oxidation [8]. Many studies explored effects of dietary phosphate addition on lipid metabolism and fatty acid oxidation. For example, Tanaka et al. [9] found that Pi deficiency increased hepatic lipid accumulation. Peri-Okonny et al. [10] reported that dietary Pi excess negatively regulates lipid synthesis in skeletal muscle. Chun et al. [11] pointed out that dietary Pi excess promotes energy expenditure via the fatty acid β-oxidation in the liver. Liu et al. [12] found that dietary phosphate reduced lipid deposition in the liver by activating Ampk pathway. However, at present, the mechanism of dietary phosphate affecting mitochondrial fatty acid oxidation is unclear in the intestine.

Multiple signaling molecules and pathways participate in the regulation of mitochondrial lipid metabolism. Among these signaling molecules, krüppel-like factor 4 (KLF4), an important zinc-finger protein, mediated the regulation of mitochondrial homeostasis and lipid metabolism by PPARα pathway [13, 14]. SIRT3, the main deacetylase localized in the mitochondria, regulates fatty acid β-oxidation in the mitochondria by regulating the acetylation of the rate-limiting enzyme long-chain acyl-CoA dehydrogenase (LCAD), which catalyzes the first step of mitochondria fatty acid oxidation [15–17]. PPARα, an important transcriptional factor, regulates mitochondrial fatty acid β-oxidation by regulating the LCAD transcriptional expression [18]. Studies also pointed out that high phosphate induced KLF4 expression [19]. Thus, we hypothesized that Klf4 may be a new potential target for dietary Pi excess inducing lipid degradation, and the Klf4-Sirt3/Pparα-Lcad pathway may be a potential regulatory pathway.

Fish have more than 30,000 species and accordingly are the most abundant vertebrate in the world. Studies pointed out that fish underwent the fish-specific genome duplication (FSGD) events during the evolution, compared with terrestrial animals^20^. As some duplicated genes will evolve the new functions, new regulatory mechanisms have emerged [20]. Yellow catfish *Pelteobagrus fulvidraco*, a widely distributed freshwater teleost fish species in China and several other Asian countries, is economically important for aquaculture because of its good fillet quality and easy culture. However, under the intensive aquaculture, yellow catfish is easy to deposit excessive lipid in the intestine, which influences its growth performance and health. Gong et al. [21] published the whole-genome sequence information of yellow catfish and found the FSGD for the fish species. Therefore, using yellow catfish as the model, our study was performed to determine the mechanism of dietary Pi on intestinal lipid degradation. We found that dietary Pi addition induced lipid degradation via the mitochondrial fatty acid β-oxidation and Klf4-Sirt3/Pparα-Lcad pathway, which provided potential preventive targets against excess intestinal lipid deposition.

## Materials and methods

### Feed formulation and yellow catfish culture

Three experimental diets were formulated with NaH_2_PO_4_·2H_2_O at the addition levels of 0 (the control, low phosphorus, LP), 1.5% (intermediate phosphorus, IP) and 3.0% (high phosphorus, HP) (Additional file 1: Table S1), based on the study by Luo et al. [22]. Phosphorus contents of diets and intestine were determined using the molybdovanadate method (spectrophotometry) as described previously [23]. Final dietary Pi contents were determined to be 3.22, 6.47 and 7.99 g Pi kg^− 1^ for the low-, intermediate- and high-Pi diets, respectively. High dietary Pi was thought to meet Pi requirement for yellow catfish [22].

Yellow catfish were obtained from a local fish farm (Wuhan, China), and cultured in indoors circular fiberglass tanks for a 14-day acclimatization. After the acclimatization, 270 juvenile yellow catfish (mean initial weight: 3.25 ± 0.01 g/fish, mean ± SEM) were stocked in nine tanks and there were 30 fish each tank (300-L water volume). Each experimental diet was fed to triplicate tanks randomly. During the feeding, yellow catfish were fed to satiation twice daily (8:00 a.m. and 4:00 p.m., respectively). All tanks were placed under indoors controlled conditions with a 12-h day/night cycle. The feeding experiment continued for 8 weeks. Water quality were monitored twice one week, and the values were followed: water temperature, 28.0 ± 1.0 °C; pH, 7.89 ± 0.15; dissolved oxygen, 6.01 ± 0.12 mg L^− 1^; NH_4_-N, 0.12 ± 0.03 mg L^− 1^, respectively.

At the end of the feeding experiment, all yellow catfish were fasted overnight. They were then euthanized (MS222 at 100 mg/l), counted, measured and weighed to determine the survival, weight gain (WG), specific growth rate (SGR) and condition factor (CF). The intestinal samples were randomly selected from two fish from each tank and used for H&E staining, Oil Red O staining and TEM analysis. Another three fish were collected randomly from each tank and their whole intestine tracts were used for the determination of phosphorus concentration. Another three fish from each tank were sampled for the intestine and used for analyzing TG contents, enzyme activities, the contents of NEFA, ATP, NAD^+^ and NADH, and the NAD^+^/NADH ratio. The intestinal tissues from another four fish each tank were sampled for the analysis of gene and protein expression. Before the analysis, all samples were fast frozen in liquid nitrogen and stored at − 80 °C freezer.

### Cell culture and incubations

Primary intestinal epithelial cells (IECs) were isolated from yellow catfish intestine by two-step collagenase-protease enzymatic digestion, as described elsewhere [24]. The IECs were cultured in Dulbecco’s modified Eagle’s medium (DMEM) containing 10% FBS (fetal bovine serum), 25 µg/ml gentamycin, 100 µg/ml streptomycin, 100 U/ml penicillin, 5 µg/ml transferrin, 10 ng/ml EGF (epidermal growth factor), and 2% D-sorbitol (S-DMEM) in 5% CO_2_ at 28 °C. The analytical protocols were showed in Additional file 1: Text S1. The HEK293T cells were obtained from the Cell Resource Center of Huazhong Agricultural University and cultured in DMEM containing100 U/ml penicillin, 10% FBS and 100 µg/ml streptomycin in 5% CO_2_ at 37 °C. To investigate the influences of Pi incubation on lipid metabolism in the IECs of yellow catfish, we used 0.9 mM Pi (the control) and 2.0 mM Pi (Pi-treated group) to incubate the primary IECs, respectively. The incubation continued for 48 h. We analyzed the parameters related to lipid metabolism (such as TG contents, enzyme activities, contents of NEFA, ATP, NAD^+^ and NADH), and the mRNA and protein expression of mitochondrial relevant genes (such as Klf4, Sirt3, Pparα and Lcad).

### Hematoxylin and eosin (H&E) and oil red O (ORO) staining, transmission electron microscope (TEM) analysis, and BODIPY 493/503 staining

We performed the H&E and ORO staining, and TEM observation for the intestinal tissues, as described previously [25]. In brief, fresh intestine tissues were rapidly isolated and fixed with the 4% paraformaldehyde in phosphate-buffered saline (PBS, pH 7.4) and embedded in the paraffin. Then the samples were stained with H&E and ORO, respectively, and used for microscopic observation. The relative areas for intestinal villus structure in H&E staining and lipid droplets (LDs) in ORO staining were quantified via the Image J software (NIH, Bethesda, MD, USA). The intestinal villus height, muscular thickness and microvilli length were analyzed using the image analysis program Image J, as described by Dong et al. [26]. Samples for TEM observation was conducted, based on the protocols by Wei et al. [27]. and the TEM observation was performed via the FEI Tecnai G^2^ 20 TWIN TEM (Leica Microsystems, Wetzlar, Germany).

The BODIPY 493/503 (D3922; Thermo Fisher Scientific, USA) staining was performed for primary IECs, as described by Wei et al. [27]. The laser scanning confocal microscope (Leica Microsystems, Germany) was used to visualize their fluorescence intensities after the BODIPY 493/503 staining, and the Image J software (NIH, Bethesda, MD, USA) was used to quantify the LDs area (green dots).

### Enzymatic activity, TG and NEFA contents measurement

The carnitine palmitoyl-transferase 1 (CPT1) activity was determined based on the measurement of the initial CoA-SH formation by the 5, 5’-dithio–bis– (2–nitrobenzoic acid) (DTNB) reaction from palmitoyl–CoA by mitochondrial samples with L–carnitine at 412 nm and the protocols followed Wei et al. [27]. One unit of enzyme activity (IU) was defined as the amount of enzyme that converted 1 µM of the substrate to product per min, and expressed as units per milligram (mg) of soluble protein. The TG and NEFA contents were analyzed with the commercial kit (Nanjing Jian Cheng Bioengineering Institute, Nanjing, China). Protein concentrations were measured with the BCA protein assay kit (P0012S, Beyotime, China).

### Measurement of ATP, NAD^+^, NADH and NAD^+^/NADH ratio

The ATP quantification kit (S0026, Beyotime Biotechnology, China) was used to analyze the ATP content, based on protocols described in Zhang et al. [28]. Briefly, the samples were washed with the ice-cold PBS and scraped into the TE buffer to boil for 5 min. Then, the samples were collected into 1.5 mL centrifuge tube. ATP quantification kit was used with 10 mL of supernatant and the standard curve was generated using purified ATP. The NAD^+^ and NADH contents were measured via the NAD^+^/NADH quantification kit (S0175, Beyotime Biotechnology, China). Protein concentrations were analyzed the BCA protein assay kit (P0012S, Beyotime Biotechnology, China).

### RNA extraction and quantitative real-time PCR (qPCR)

The qPCR assays were performed with SYBR Green PCR Master Mix (Applied Biosystems) and experimental protocols were described in Zhang et al. [28]. Ten housekeeping genes (*β-actin, 18s rRNA, hprt, ubce, gapdh, tuba, b2m, rpl7, tbp* and *elfa*) were measured as endogenous controls for their mRNA expression, and the best combination of two genes were analyzed by geNorm (https://genorm.cmgg.be/) [29]. The 2^−ΔΔCt^ method was used to analyze the relative fold changes. The primers are listed in Additional file 1: Table S2.

### Construction of reporter plasmids and cell transfections

To explore the underlying mechanisms for Pi-induced lipolysis, we constructed the Klf4, Sirt3, Lcad, Errα and Pgc1α expression vectors based on the published protocols [12]. We subcloned the open reading frames (ORFs) of Sirt3 and Pgc1α sequences into the pcDNA3.1 (+) vector with the HA-tag at the N-terminus, respectively. The open reading frame (ORF) of Errα sequences was also subcloned into the pcDNA3.1 (+) vector with the Myc-tag at the N-terminus. The ORFs of Klf4 and Lcad sequences were subcloned into the pcDNA3.1 (+) vector with Flag-tag sequence inserted at the N-terminus, respectively. The primers are given in Additional file 1: Table S3. We performed the transient transfection of the plasmids into 293T cells via the Lipofectamine 2000 (Invitrogen), based on the protocols mentioned above.

For Klf4-knockdown experiment, three *klf4*-siRNA sequences were transfected with the Entranster^TM^-R4000 Reagent kit (Engreen Biosystem, 4000-4) to primary IECs, respectively. The siRNA knockdown sequence was given in Additional file 1: Table S4. The knockdown efficiencies of the *klf4*-siRNA sequences were determined by the qPCR and Western blot analysis, respectively. The siRNA-1164 was chosen to perform the *klf4*-knockdown experiment because of its high efficiency for inhibiting the *klf4* expression. After primary IECs were transfected with NC’s (negative control) siRNA and *klf4* siRNA, cells were cultured for 48 h with two treatments: the control (without extra Pi addition) and Pi incubation (2mM), respectively.

### Luciferase reporter assays

Based on the hi-TAIL PCR (high-efficiency thermal asymmetric interlaced-PCR) method [30], the *sirt3* promoter-luciferase (− 1576/+44 bp), *pparα* promoter-luciferase (− 837/+63 bp) and *lcad* promoter-luciferase (− 1618/+20 bp) construct and their mutants were built, based on the protocols described in Xu et al [30]. Briefly, the transcription start sites (TSS) and 5’ cDNA sequences were identified by RLM-5’RACE (RNA ligase-mediated rapid amplification of 5’ cDNA ends) method. The mutant promoter reporters (Mut) were performed according to the manufacture instruction with the Quick-Change Site-Directed Mutagenesis Kit (Vazyme), based on predictions from the JASPAR database (http://jaspar.genereg.net/). Finally, the plasmids containing the promoter sequences were subcloned into Sac I/Hind III sites of pGL3-basic vector. The specific primers were given in Additional file 1: Table S5 and S6. The luciferase activity was measured with the Dual Luciferase Reporter Assay System (Promega, Minneapolis, USA), based on the protocols in Xu et al [30]. Briefly, 293T cells were maintained at 5% CO_2_ and 37 °C in the DMEM containing 10% FBS. The 400 ng reporter plasmids were co-transfected into the 293T cells and the 20 ng pRL-TK plasmids was used as the control. After 4 h, the medium was replaced by the DMEM or DMEM with 2mM Pi incubation. After 24 h incubation, cells were collected to analyze the relative luciferase activities.

### Electrophoretic mobility shift assay (EMSA)

The EMSA was performed to analyze the direct binding sites at the promoters, as described previously [30]. All oligonucleotide sequences were shown in Additional file 1: Table S7. Briefly, the EMSA was performed using an Invitrogen kit with SYBR Green (E33075; Invitrogen). Nuclear proteins were extracted from the primary IECs which were incubated with or without 2 mM Pi for 24 h, using a cytoplasmic and nuclear extraction kit (Viagene Biotech, China). The Bio-Rad gel imaging system was used to analyze the reactions.

### Western blot analysis, immunoprecipitation assays and immunofluorescence staining

Western blot analysis was conducted to explore the protein expression of Slc34a2, Ucp2, Klf4, Sirt3, Pparα and Lcad, based on the protocols in Liu et al. [12]. In brief, intestinal tissues and primary IECs samples were homogenized in ice-cold radioimmunoprecipitation assay buffer (Thermo Fisher Scientific, USA) supplemented with protease and deacetylase inhibitors (Roche, Indianapolis, IN, USA). The lysates were incubated at 4 °C for 30 min and centrifuged at 12,000 g for 15 min. The protein concentrations of samples were determined via the BCA protein assay kit (Beyotime, China). Proteins sample were separated on the 10% or 12% SDS–PAGE polyacrylamide gels, and then transferred to PVDF (polyvinylidene fluoride) membranes (Millipore, USA). The membranes were blocked with 5% defatted milk in TBST solution (Tris-buffered saline with Tween) at room temperature for 2 h and incubated overnight at 4 °C with primary antibodies below: anti-KLF4 (Cell Signaling Technology, 4038), anti-SIRT3 (Cell Signaling Technology, 2627), anti-LCAD (Proteintech, 17526-1-AP, USA), anti-PPARα (Proteintech, 15540-1-AP), anti-HA tag (Abcam, ab18181), anti-Flag tag (Abcam, ab1162), anti-GAPDH (CST, 2118) and anti-acetyl lysine (Abcam, ab21623). After washing with TBST solution, the membranes were incubated with horseradish peroxidase-conjugated goat anti-rabbit or anti-mouse IgG secondary antibody (1:5000, HuaBio, China) at room temperature for 2 h. Next, the protein bands were identified by the Odyssey Infrared Fluorescent Western Blots Imaging System (Li-Cor Bioscience) with the enhanced ECL substrate (Bio-Rad). The protein bands were quantified using the ImageJ software (version 2.0.0, USA). The protein expression was normalized to endogenous GAPDH.

The immunoprecipitation assay was conducted according to our publication [27]. The HA-Sirt3 vector was co-transfected into the 293T cells together with Flag-Lcad, Flag-Klf4, and Myc-Errα vectors, respectively. The empty vectors were co-transfected as the control. After 24 h, the cells were collected and the protein was extracted. The Flag monoclonal antibodies, HA monoclonal antibodies and Myc monoclonal antibodies were used for the IP testing, followed by Western blot detection using anti-Flag tag, anti-HA tag and anti-Myc antibodies, respectively.

The immunofluorescence staining was conducted, as previously described [27]. Briefly, the cells were fixed in 4% PFA (paraformaldehyde) solution at room temperature for 20 min, washed twice in ice-cold PBS, permeabilized in PBS-Triton and incubated with the specific primary antibody mentioned above overnight at 4 °C. Next day, the cells were washed in ice-cold PBS. They are incubated with the secondary antibody for 1 h. We used DAPI (Invitrogen) to stain the nucleus of hepatocytes. The images were acquired with the laser scanning confocal microscope (Leica, Wetzlar, Germany), and quantified by the Image J software.

### Statistical analysis

All data were presented as the mean ± SEM (n ≥ 3). The statistical analyses were performed by the software SPSS version 19 ((IBM, Armonk, NY, USA). The data were analyzed for the normality with the Kolmogorov–Smirnov test. The Bartlett’s test was used to analyze the homogeneity of the variances among the treatments. The Student’s T test was utilized to analyze these data between two treatments. The one-way ANOVA and Duncan’s multiple range test were utilized to analyze the experimental data among more than three treatments. The GraphPad Prism 8.0 software (GraphPad Software, USA) was used to draw figures. *P*-values < 0.05 were considered significant.

## Results

### Expt. 1: in vivo study

#### Growth performance and feed utilization

The survival was not influenced significantly by dietary phosphorus levels (Additional file 1: Table S8). Compared to LP group, weight gain (WG), specific growth rate (SGR) were significantly increased in IP and HP groups. Feed conversion rate (FCR) was lower in HP group than those in LP and IP groups. Feed intake (FI) and condition factor (CF) showed no significant differences among the three treatments.

#### HP diet influenced intestinal histology and increased intestinal phosphate transport

**Fig. 1:**
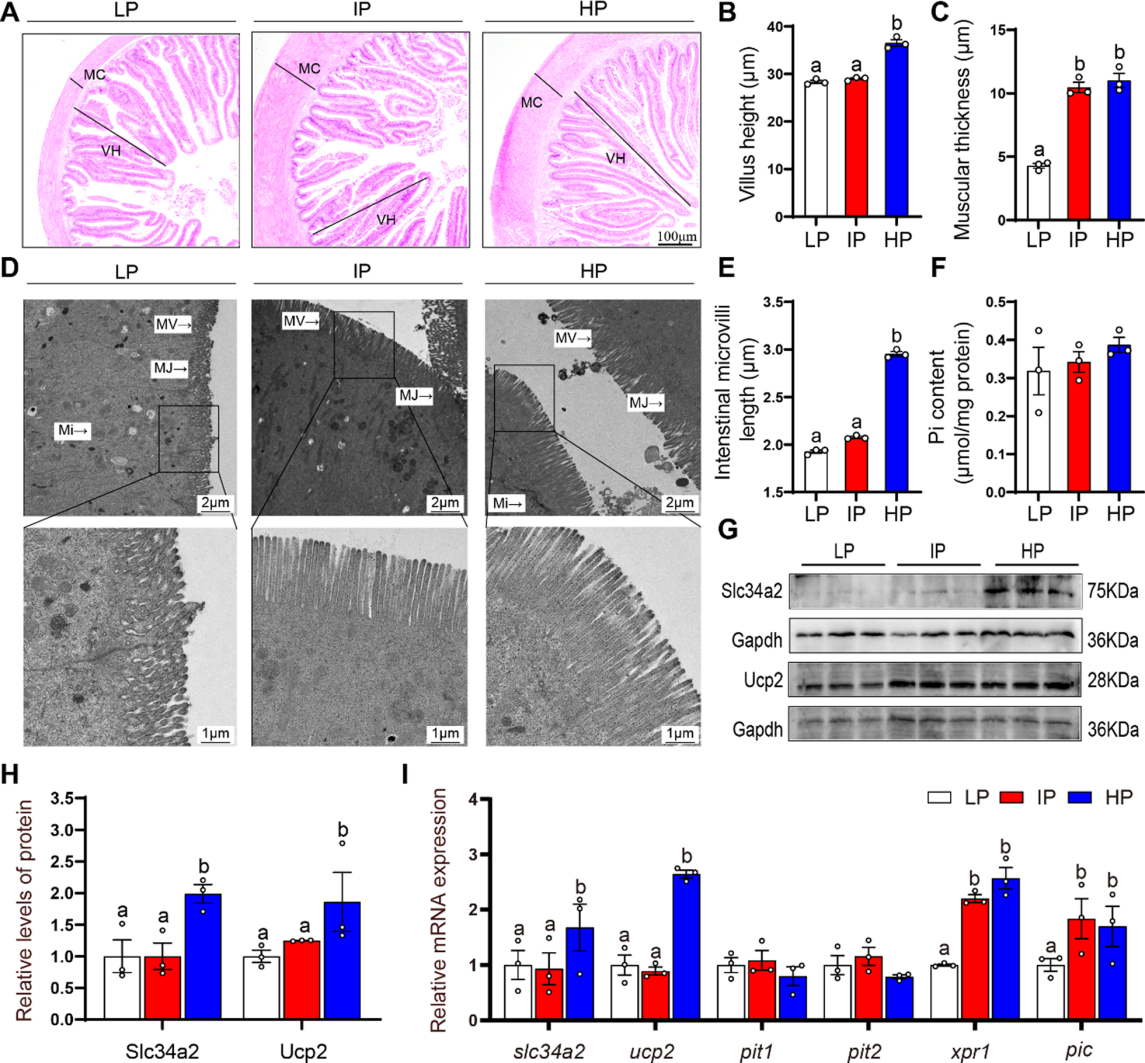
High dietary phosphorus influenced the ultrastructure and phosphorus absorption in the intestinal tissues of yellow catfish. **A** Representative images of intestinal H&E staining. Scale bar, 100 μm. **B** Villus height. **C** Muscular thickness. **D** Intestinal microvilli ultrastructure. Scale bar, 2 μm. **E** Quantification of intestinal microvilli length. **F** Pi content. **G**, **H** Western blot and quantification analysis of SLC34A2 and UCP2 protein expression. **I** The mRNA levels of genes related to Pi transporter. Labeled means without a common letter differ, *P* < 0.05 (1-factor ANOVA, Duncan post hoc test). MJ, micro-tight junction; MV, microvilli; Mi, mitochondria

The intestine is an important organ for the absorption of inorganic phosphorus. To understand the effects of dietary phosphate addition influencing intestinal structure and phosphate transport, we analyzed several essential indicators. Compared to low dietary phosphorus group, high dietary phosphorus showed a more integral intestinal structure, such as the higher intestinal villus (Fig. 1A, B), the thicker muscular layers (Fig. 1C), and longer and more integral intestinal microvilli (Fig. 1D, E). The intestinal Pi content was not significantly influenced by dietary Pi addition (Fig. 1F). Compared to the low and intermediate dietary phosphate addition, high dietary phosphate levels up-regulated the protein expression of Slc34A2 and Ucp2 (Fig. 1G, H), and increased the *slc34a2* and *ucp2* mRNA expression (Fig. 1I). The *xpr1* and *pic* mRNA expression was higher in the IP and HP groups than those in the LP group. The *pit1* and *pit2* mRNA levels was not significantly influenced by dietary Pi addition (Fig. 1I). Thus, dietary phosphate addition was essential for the intestinal structure, and high dietary Pi levels activated the intestinal phosphate absorption and excretion, and accordingly maintained the intestinal phosphate homeostasis.

#### HP diet induced intestinal lipid degradation via mitochondrial fatty acid β-oxidation

**Fig. 2:**
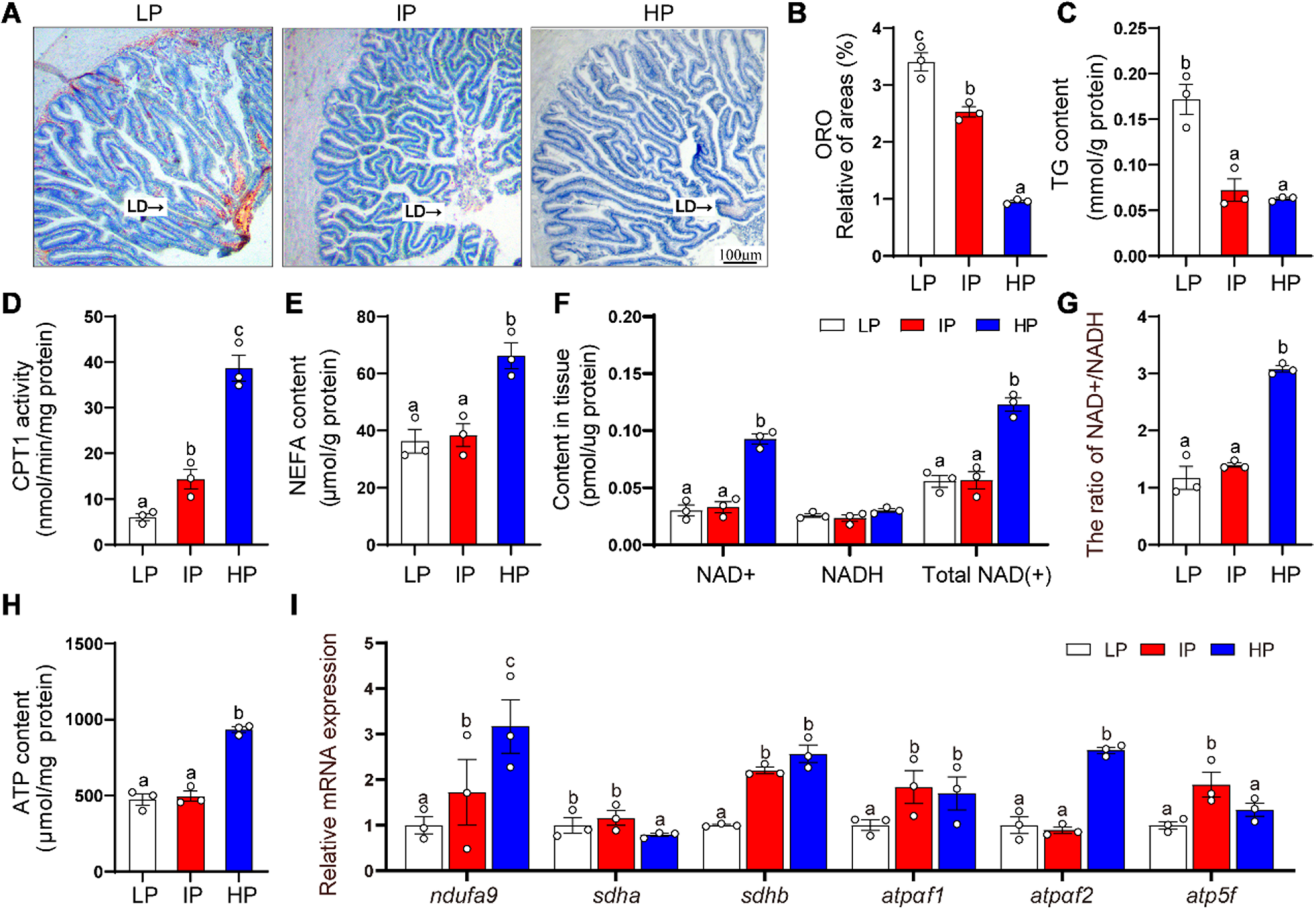
High dietary phosphorus influenced the intestinal histology, increased lipolysis and affected the intestinal mitochondrial homeostasis in yellow catfish. **A** Representative images of intestinal Oil Red O staining. Scale bar, 100 μm. **B** Relative areas for LDs in ORO staining. **C** TG content. **D** CPT1 activities. **E** NEFA content. **F** NAD^+^, NADH and total NAD contents. **G** NAD^+^/NADH ratio. **H** ATP content. **I** The mRNA levels of genes related to ATP synthesis. Labeled means without a common letter differ, *P* < 0.05 (1-factor ANOVA, Duncan post hoc test). LD, lipid droplet; MC, Muscular thickness; VH, Villi height

In order to investigate the effects of dietary phosphorus addition on intestinal lipid content, we measured key indicators of lipid metabolism and mitochondrial homeostasis. The amount of lipid droplets (LDs) (Fig. 2A, B) and the TG content (Fig. 2C) declined, but CPT I activity increased (Fig. 2D) with increasing dietary phosphorus levels. The NEFA content was higher significantly in the HP group than those in the LP and IP groups (Fig. 2E). NAD^+^ is at center of OXPHOS, and mitochondrial function is critical for the NAD^+^/NADH balance [31]. The contents of NAD^+^ and total NAD^+^ (Fig. 2F), the ratio of NAD^+^/NADH (Fig. 2G) were higher in the HP group than those in the LP and IP groups. In contrast, NADH content was not significantly influenced by dietary Pi levels. ATP content was considered as an important marker to reflect mitochondrial function [7]. Herein, HP groups significantly increased the intestinal ATP content (Fig. 2H). The mRNA levels of various subunits of OXPHOS complexes I (*ndufa9*), complexes II (*sdha, sdhb*) and ATP synthase genes (*atpαf1, atpαf1* and *atp5f*) were analyzed [32]. The *ndufa9* mRNA expression increased with increasing dietary phosphorus levels (Fig. 2I). The mRNA expression of *sdhb* and *atpaf1* was higher in the IP and HP groups than those in the LP group (Fig. 2I). The *sdha* mRNA expression was lower but the *atpaf2* mRNA expression was higher in the HP group than those in the LP and IP groups. The *atp5f* mRNA level was higher in the IP group than those in the LP and HP groups. Taken together, dietary Pi addition reduced lipid deposition, increased lipolysis and affected mitochondrial homeostasis.

**Fig. 3:**
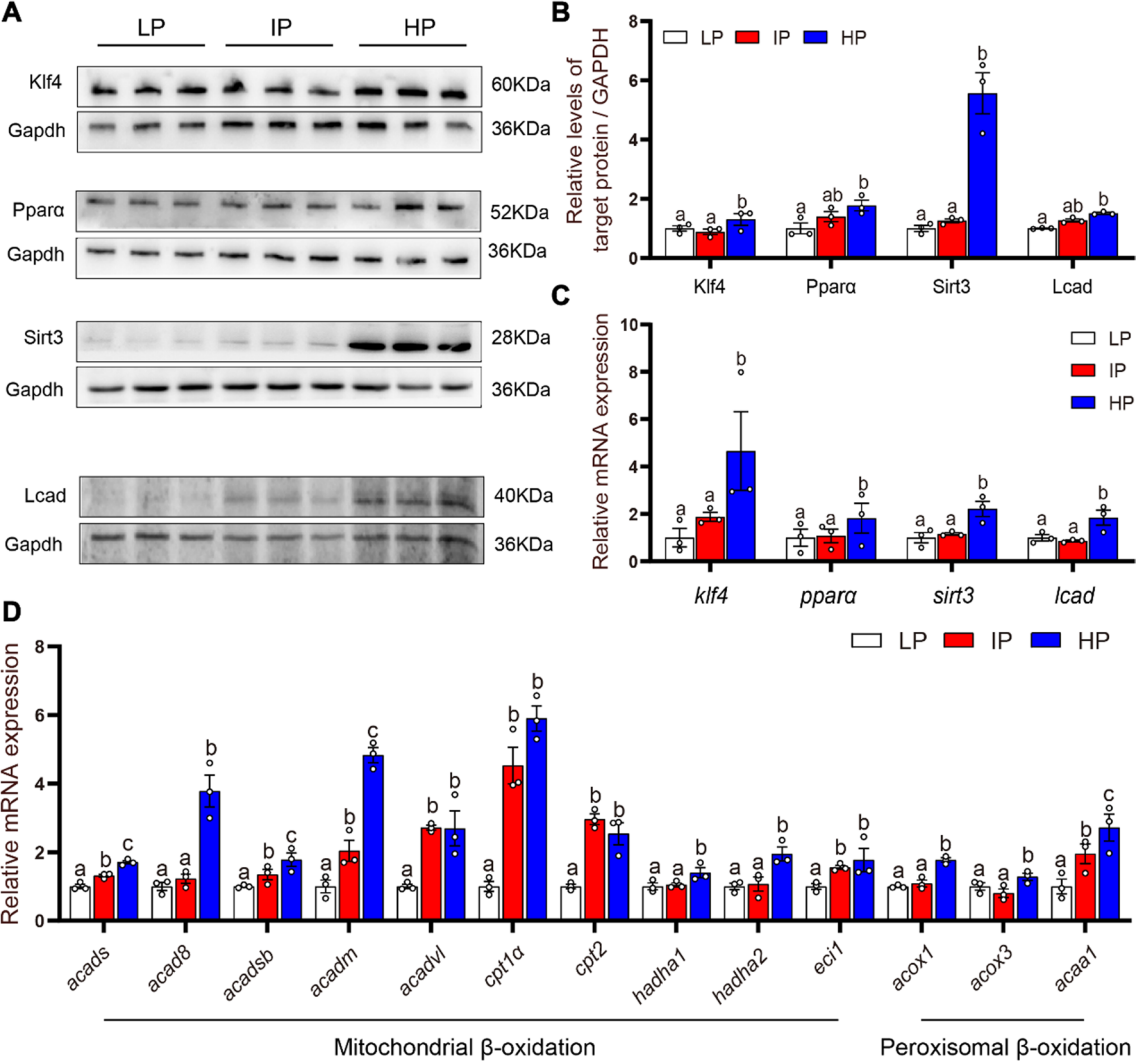
High dietary phosphorus influenced mitochondrial fatty acid β-oxidation in the intestine tissues of yellow catfish. **A**, **B** Protein expressions of KLF4, PPARα, SIRT3, and LCAD after Western blot analysis (n = 3). **C** The mRNA levels of *klf4, pparα, sirt3* and *lcad*. **D** The mRNA levels of mitochondria fatty acid β-oxidation-related genes (n = 3). Labeled means without a common letter differ, *P* < 0.05 (1-factor ANOVA, Duncan post hoc test)

To further explore the potential mechanisms, the key proteins relevant with mitochondrial homeostasis were analyzed. Klf4, Pparα and Sirt3 are critical regulators of mitochondrial homeostasis [18, 33, 34]. LCAD, a rate-limiting enzyme, participates in the mitochondrial fatty acid β-oxidation. Studies suggested that LCAD was regulated by SIRT3 and PPARα [35. 36]. Here, compared to the LP and IP groups, high dietary phosphorus up-regulated the mRNA and protein expression of Klf4, Pparα, Sirt3 and Lcad (Fig. 3A–C). The mRNA expression of the mitochondrial fatty acid β-oxidation genes (*acads*, *acad8*, *acadsb*, *acadm*, *hadha1*, *hadha2, cpt1a*, *cpt2* and *eci1*) and peroxisomal β-oxidation genes (*acox1*, *acox3* and *acca1*) was significantly higher in the HP group than those in the LP group (Fig. 3D). Taken together, high dietary phosphorus addition activated the fatty acid β-oxidation in the mitochondria and accordingly induced intestinal lipid degradation.

### Experiment 2: in vitro study

#### Pi incubation reduced TG contents and increased lipolysis in the IECs

**Fig. 4:**
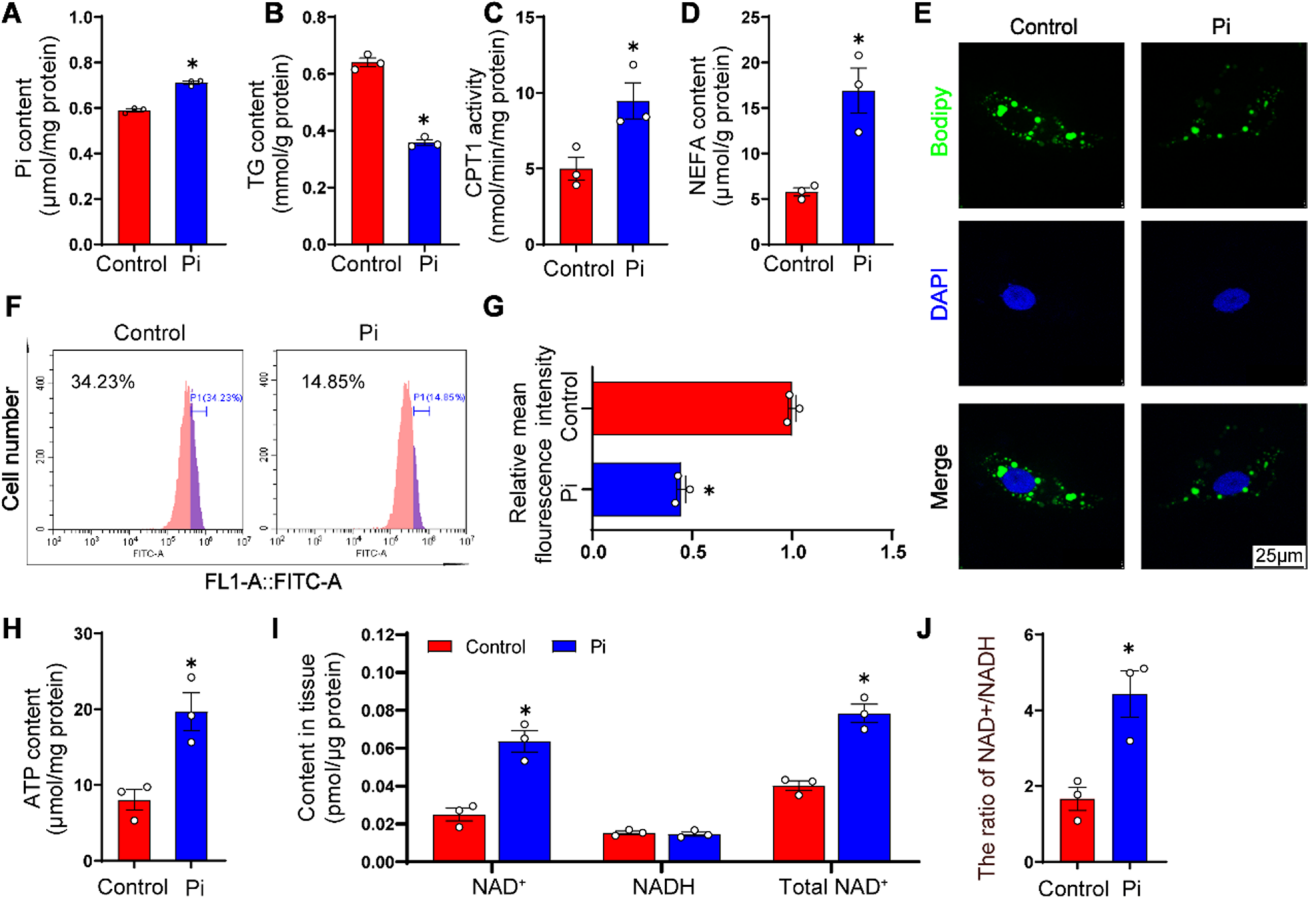
Phosphorus incubation induced lipolysis and influenced mitochondrial homeostasis in the intestinal epithelial cells (IECs) of yellow catfish. **A** Pi content. **B** TG content. **C** CPT1 activities. **D** NEFA content. **E** Representative confocal microscopy images of primary IECs in yellow catfish with BODIPY 493/503 staining. Scale bar, 25 μm. **F** Presence of LDs stained with BODIPY 493/503 demonstrated by flow cytometry. **G** Lipid content quantified by flow cytometric analysis of FL1 (bule) mean fluorescence intensity with Bodipy 493/503 staining. **H** ATP content. **I** NAD^+^, NADH and total NAD contents. **J** NAD+/NADH ratio. All data were expressed as mean ± S.E.M. (n = 3). *P < 0.05, compared with the control

To elucidate the mechanisms of high dietary phosphate addition increasing lipolysis, we conducted several in vitro experiments. The MTT assay showed that Pi of not more than 2 mM did not adversely influence the viability of the IECs of yellow catfish (Additional file 1: Fig. S1). Compared to the control, Pi incubation increased intracellular Pi content (Fig. 4A), reduced TG content (Fig. 4B), increased CPT 1 activity (Fig. 4C) and intracellular NEFA content (Fig. 4D). Meanwhile, the LDs amounts were quantified by the flow cytometric analysis and imaged by the confocal microscopy after the BODIPY 493/503 staining (Fig. 4E, F). The results indicated that Pi incubation decreased LDs accumulation in the IECs (Fig. 4G). Compared to the control, Pi treatment increased ATP (Fig. 4H), NAD^+^ and total NAD^+^ contents, and the ratio of NAD^+^/NADH (Fig. 4I, J). Taken together, Pi incubation induced lipid degradation and affected mitochondrial homeostasis in the IECs of yellow catfish.

#### Pi incubation activated KLF4-SIRT3 and KLF4-PPARα pathway in the IECs

**Fig. 5:**
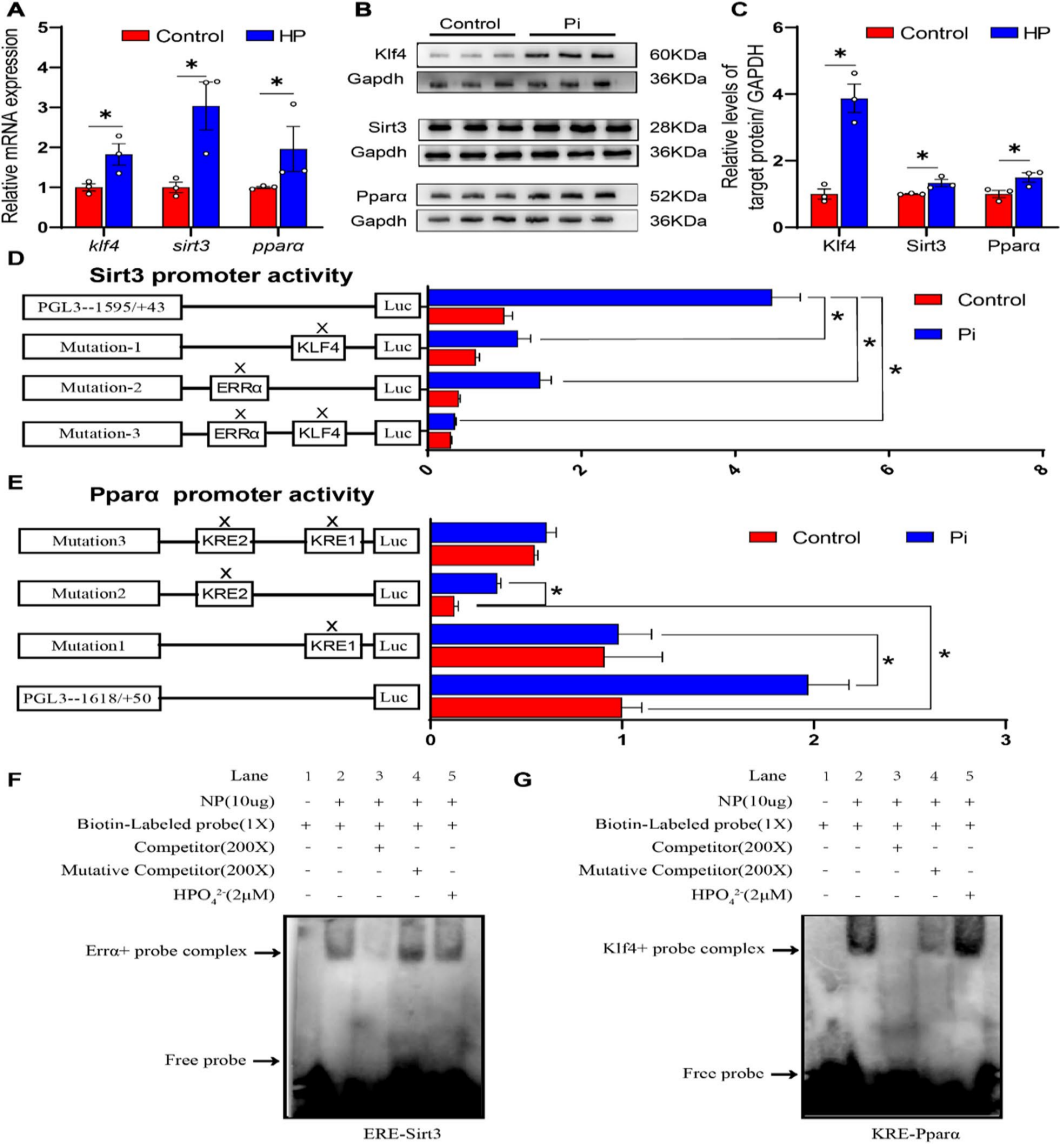
Phosphorus incubation activated KLF4-SIRT3 and KLF4-PPARα pathway in the intestinal epithelial cells (IECs) of yellow catfish. **A** The mRNA levels of *klf4*, *sirt3*, *pparα*. **B**, **C** Western blot analysis and quantification analysis of KLF4, SIRT3, PPARα protein levels. **D** Relative luciferase activity of *sirt3* promoter. **E** Relative luciferase activity of *pparα* promoter. **F** EMSA of putative SIRT3 binding sequences (ERE). **G** EMSA of putative PPARα binding sequences (KRE). All data were expressed as mean ± S.E.M. (n = 3). P value was calculated by Student’s t tests. *P < 0.05, compared with control. IP, immunoprecipitation; NS, non-significance

Klf4, Sirt3 and Pparα are key proteins who mediate the mitochondrial fatty acid β-oxidation [13, 15, 18]. Compared to the control, Pi treatment up-regulated the mRNA and protein expression of *klf4*, *sirt3* and *pparα* (Fig. 5A–C). Pi-induced increase of KLF protein expression was confirmed further by the immunofluorescent staining (Additional file 1: Fig. S2).

To elucidate whether *klf4* regulated the expression of *sirt3* at the transcription level, we cloned the sequences of *sirt3* promoters, as shown in Additional file 1: Fig. S3. Studies suggested that KLF4 regulates target proteins via the ERRα/PGC-1α pathway [13], and ERRα/PGC-1α-SIRT3 pathway regulates the expression of mitochondrial protein [37]. Based on JASPAR predictions, we detected the putative binding sites of KLF4 and ERRα in the *sirt3* promoter (Additional file 1: Fig. S4). For the analysis of *sirt3* promoter, the disruption of the − 406/−419 bp Klf4 binding site (KRE), and − 1444/−1457 bp Errα binding site (ERE) significantly attenuated the Pi-induced increase of *sirt3* promoter activity (Fig. 5D). Next, we sought to determine whether the direct binding of Klf4 to KRE and ERE were required for the activation of *sirt3* promoter. We found that overexpression of Klf4 with Errα and Pgc1α vectors significantly increased the *sirt3* promoter activity, compared with single overexpression of Klf4 vector or Errα overexpression with Pgc1α vectors. However, under the overexpression of Klf4 with Errα and Pgc1α vectors, the disruption of the − 406/−419 bp Klf4 binding site (KRE) did not significantly change the *sirt3* promoter activity, but the deletion of − 1444/−1457 bp Errα binding site (ERE) significantly attenuated the overexpression of Klf4 with ERRα and Pgc1α vectors-induced increase of the activity of *sirt3* promoter (Additional file 1: Fig. S5). To further explore whether Klf4 and Errα can bind to Sirt3 transcription module, the Klf4 co-precipitated with Sirt3 in the 293T cells with Flag-Klf4 and HA-Sirt3 overexpression (Additional file 1: Fig. S6A). The Errα co-precipitated with Sirt3 in the 293T cells with Myc-Errα and HA-Sirt3 overexpression (Fig. S6B). To elucidate whether *klf4* regulated the expression of *pparα* at the transcription level, we cloned the sequences of *pparα* promoters, as shown in Additional file 1: Fig. S7. We predicted the Klf4 response elements 1 (KRE1) and Klf4 response elements 2 (KRE2) located at − 474 to − 453 bp and − 534 to − 515 of *pparα* promoter, respectively (Additional file 1: Fig. S8). The KRE1 mutation vector significantly attenuated Pi-induced increment of activity of the *pparα* promoter. Without the Pi incubation, the KRE2 mutation vector down-regulated the basal activity of the *pparα* promoter; however, after the KRE2 site was mutated, Pi incubation increased activities of *pparα* promoter (Fig. 5E). The EMSA showed that the putative ERRα binding sites of *sirt3* promoter could bind directly with the nuclear extract, but the binding was disrupted by the unlabeled wild-type probe and restored by the mutant probe (Fig. 5F). Meanwhile, the EMSA further confirmed that the putative Klf4 binding sequences of the *pparα* promoter could bind directly with nuclear extract. The direct binding can be disrupted by the unlabeled wild-type and restored by the mutant probe (Fig. 5G). Taken together, these results indicated that Pi incubation activated Klf4-Sirt3 and Klf4-Pparα pathway in IECs.

#### Pi incubation activated SIRT3-LCAD and PPARα-LCAD pathway in the IECs

**Fig. 6:**
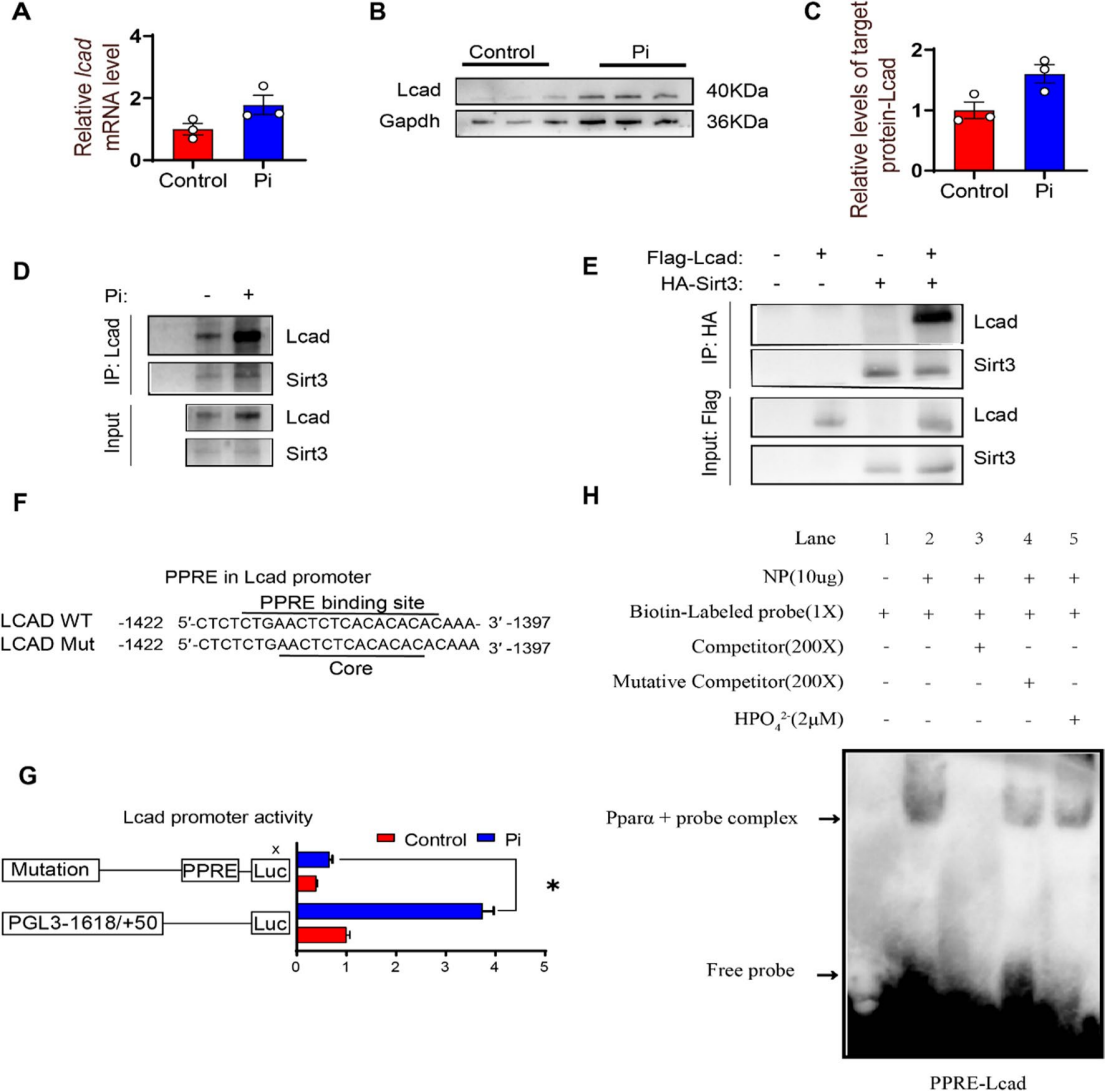
Pi incubation activated SIRT3-LCAD and PPARα-LCAD pathway in the IECs. **A** The mRNA levels of *lcad*. **B**, **C** Western blot analysis and quantification analysis of LCAD protein levels. **D** Interaction of SIRT3 with LCAD under Pi incubation. **E** Interaction of SIRT3 with LCAD, Flag-tag LCAD and HA-tag SIRT3 were transfected into 293T cells, and the interaction between SIRT3 and LCAD was determined with IP and western blot. **F** PPARα response elements (PPRE) located at − 1422 bp to − 1397 bp of *lcad* promoter. **G** Relative luciferase activity of *lcad* promoter. **H** EMSA of putative LCAD binding sequences (PPRE). *P < 0.05, compared with the control

Because studies suggested that LCAD is deacetylated by SIRT3 [34], and regulated at the transcription level by PPARα [18], we next explored the regulatory mechanism of LCAD. Compared with the control, Pi treatment increased the mRNA and protein levels of Lcad significantly (Fig. 6A–C). The immunoprecipitation analysis indicated that Pi incubation promoted the interaction between Lcad and Sirt3 (Fig. 6D). The Sirt3 coprecipitated with Lcad in the 293T cells with Flag-Lcad and HA-Sirt3 overexpression (Fig. 6E). To elucidate whether Pparα regulated the expression of *lcad* at the transcription levels, we cloned the sequences of *lcad* promoter, as shown in Fig. S9. We found the − 1422 bp to − 1397 bp Pparα-binding element (PPRE) located at *lcad* promoter (Fig. 6F). Pi incubation activated the activity of *lcad* promoter and the PPRE mutation vector attenuated the Pi-induced increase of the *lcad* promoter activity (Fig. 6G). Moreover, the EMSA further confirmed that the putative Pparα binding sequences of the *lcad* promoter could bind directly with the nuclear extract. Their binding can be disrupted by the unlabeled wild-type probe and restored by the mutant probes (Fig. 6H). Taken together, after Pi incubation, Sirt3 activated the Lcad expression, and Pparα activated the Lcad activity by binding to the PPRE on the *lcad* promoter.

#### Knockdown of KLF4 attenuated Pi-induced lipid degradation in IECs

**Fig. 7:**
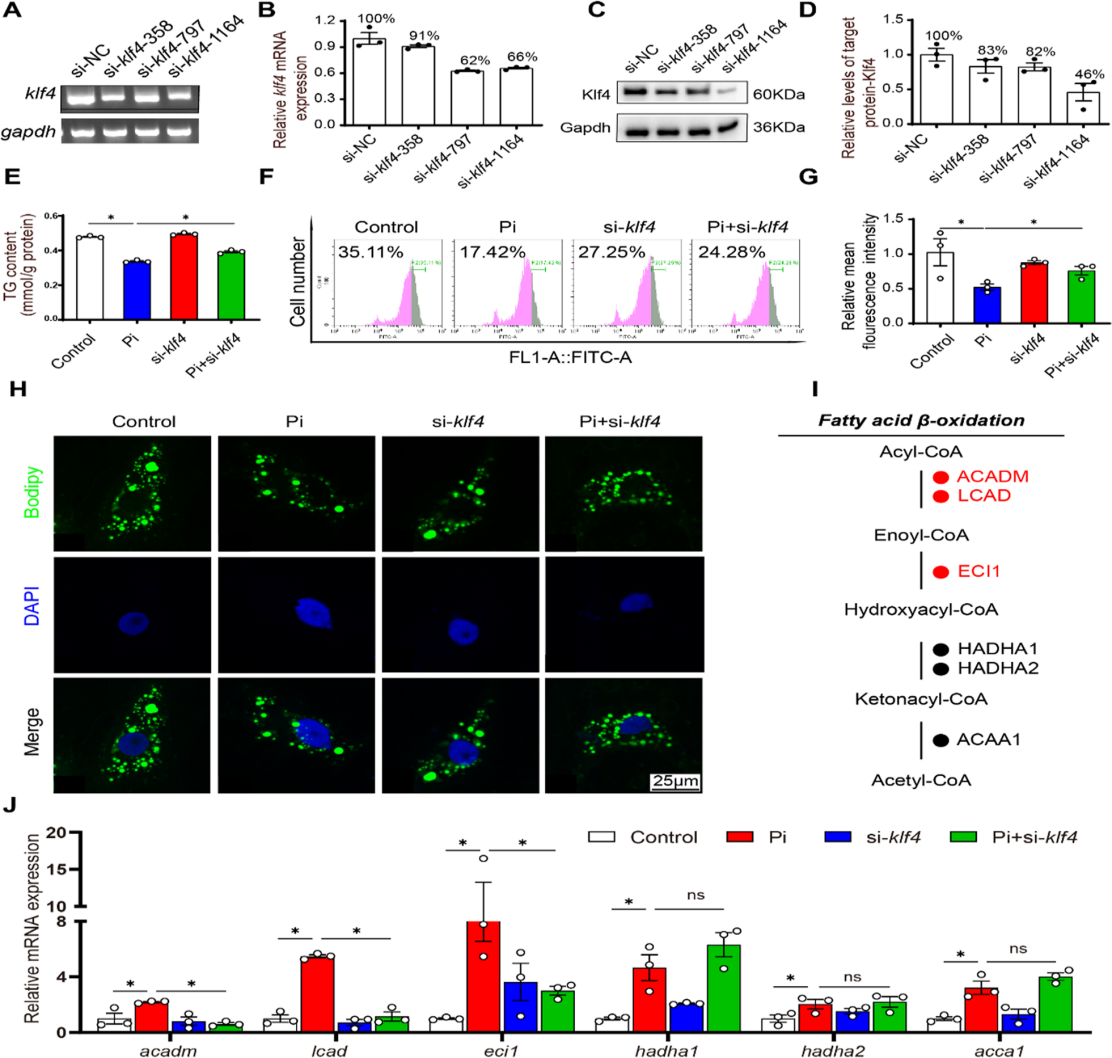
Phosphorus incubation induced lipid degradation by activating KLF4 in the IECs of yellow catfish. **A** Semi-quantitative of si-*klf4* mRNA expression. **B** Relative mRNA expression of si-*klf4*. **C**, **D** Western blot analysis and quantification analysis of siklf4-358/797/1164. **E** TG content. **F** Presence of LDs stained with Bodipy 493/503 was demonstrated by flow cytometry. **G** Lipid content was quantified by flow cytometric analysis of FL1 (green) mean fluorescence intensity with BODIPY 493/503 staining. **H** Representative confocal microscopy images of primary IECs in yellow catfish with BODIPY 493/503 staining. Bars represent 25 μm. **I**, **J** Specific enzymes in the pathway map showing mitochondria fatty acid oxidation. The mRNA levels of fatty acid β-oxidation related genes are indicated by color coding: the mRNAs with increased levels are marked in red, and with decreased or unaltered levels are marked in black. *P ≤ 0.05, **P ≤ 0.01, ***P ≤ 0.001 (One-way ANOVA with the Bonferroni post hoc test)

To investigate whether *klf4* directly regulates lipid metabolism, we used siRNA to knockdown *klf4* expression. We designed three pairs of interfering sequences to knock down *klf4* expression in IECs, and analyzed their knockdown efficiencies by semi-quantitative analysis (Fig. 7A), qPCR analysis (Fig. 7B) and western blot analysis (Fig. 7C, D). We selected the siRNA-1164 because of its high efficiency for inhibiting *klf4* expression. Compared with the control, Pi incubation reduced TG content significantly, but *klf4*-knockdown abrogated the reduction of TG content induced by Pi (Fig. 7E). Meanwhile, Pi incubation reduced the amounts of lipid droplets and *klf4* knockdown alleviated the reduction of amounts of lipid droplets induced by Pi (Fig. 7F, G). These results were further confirmed by the confocal microscopic images of the IECs stained by BODIPY 493/503 (Fig. 7H). Compared to the control, Pi treatment increased the mRNA expression of mitochondrial fatty acid oxidation (*acadm, lcad, eci1, hadha1* and *acaa1*); in contrast, *klf4-*knockdown abrogated the increase of *acadm, lcad* and *eci1* mRNA abundances induced by Pi (Fig. 7I–J).

**Fig. 8:**
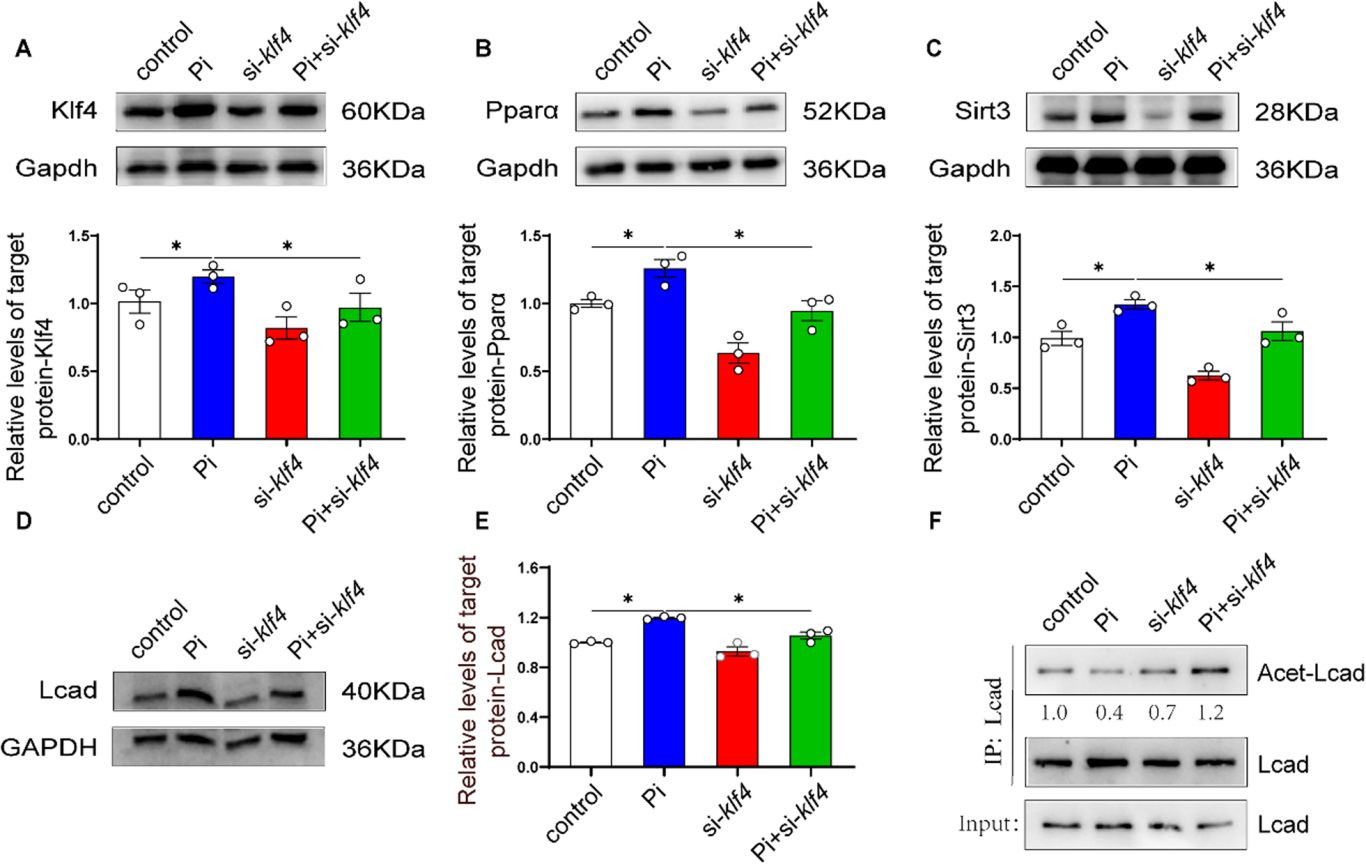
KLF4 knockdown attenuated the phosphorus-induced lipolysis in the intestinal epithelial cells (IECs) of yellow catfish. **A **Western blot analysis and quantification analysis of KLF4 protein expression. **B** Western blot analysis and quantification analysis of PPARα protein expression. **C** Western blot analysis and quantification analysis of SIRT3 protein expression. **D**, **E** Western blot analysis and quantification analysis of LCAD protein expression. **F** Quantification analysis of LCAD acetylation level. *P ≤ 0.05 (One-way ANOVA with the Bonferroni post hoc test)

To further verify whether the Klf4-Sirt3/Pparα-Lcad pathway mediates Pi-induced lipolysis, we tested their protein levels under Pi incubation after *klf4* knockdown. Pi incubation significantly increased the protein expression of Klf4, Pparα, Sirt3 and Lcad, and *klf4* knockdown attenuated the Pi-induced increase of the protein and mRNA levels of Klf4, Pparα, Sirt3 and Lcad (Fig. 8A–E). Compared to the control, Pi incubation reduced the Lcad acetylation level, and *klf4* knockdown relieved Pi-induced increment of Lcad acetylation level (Fig. 8F). Taken together, these studies demonstrated that Klf4-Sirt3/Pparα-Lcad pathway mediated high phosphate-induced lipid degradation in the intestine and IECs.

## Discussion

In our study, fish fed high dietary phosphorus increased growth performance, in agreement with other studies [12]. Compared to the low dietary phosphorus group, high dietary phosphorus group tended to reduce intestinal TG contents and up-regulated mitochondrial fatty acid oxidation, similar with other studies [10, 11]. Thus, dietary phosphate is a novel metabolic regulator, and could be useful for the potential treatment of NAFLD.

Phosphorus, an essential macro-mineral, is involved in the maintenance of intestinal homeostasis [38]. In this study, fish fed high dietary phosphate showed a more integral intestinal structure, similar to previous report [39]. The intestine is the important organ for the absorption of inorganic phosphorus. In present study, high dietary phosphate increased the Slc34a2 mRNA and protein expression and *xpr1* mRNA expression, but did not affect the *pit1* and *pit2* mRNA expression, indicating that Slc34a2 and Xpr1 might be the major phosphate transporters in intestine of yellow catfish fed high dietary phosphate. SLC34A2 was considered as the major phosphate transporter that mediates Pi reabsorption, and Xpr1 was the major phosphate exporter in mammals [1, 5]. Similarly, studies suggested that SLC34A2 expression was increased in mammals fed high phosphate diet [1, 2]. The mitochondrial PIC and UCP2 can transport inorganic phosphate into the mitochondrial matrix [3, 4]. Here, for the first time, we found that dietary high phosphate intake up-regulated the Ucp2 protein expression, and *ucp2* and *pic* mRNA expression in the intestine of yellow catfish. Taken together, our data demonstrated that high dietary phosphate increased phosphate transport and export via Slc34a2 and Xpr1, and increased intracellular Pi transport to mitochondria via Pic and Ucp2 in the intestine of yellow catfish, which help to maintain the Pi homeostasis in the intestine, as observed here.

Studies reported that high dietary phosphorus increased lipid degradation in the liver [10, 12], but effects and mechanisms of dietary phosphorus intake on lipid metabolism remain largely unknown in the intestine. Here, for the first time, we observed that high levels of dietary phosphate and Pi incubation reduced TG content but increased intracellular NEFA content in the intestine and primary IECs, suggesting the increase of TG breakdown from lipid droplets, as observed by Wei et al. [25]. Thus, our data demonstrated that high dietary phosphate and Pi incubation induced lipid degradation in the intestine. Mitochondria is the main energy-supplying organelle in cells and produces ATP via OXPHOS [8, 40]. NAD is a vital cofactor involved in ATP production via mitochondrial fatty acid β-oxidation [7]. Here, we found that high levels of dietary phosphorus and Pi incubation significantly increased ATP and NAD^+^ contents, indicating the activation of mitochondrial fatty acid β-oxidation. The *cpt1a* catalyzes the import of long-chain fatty acids into the mitochondria, and the *cpt2* reconverts acyl-carnitines back into the respective acyl-CoA esters that undergo mitochondrial β-oxidation [29]. In the present study, high levels of dietary phosphate and Pi incubation activated the CPT1 enzyme and *cpt1a* and *cpt2* mRNA expression, further suggesting the activation of β-oxidation. Acyl-CoA dehydrogenase family member 8 (*acad8*), short-chain specific acyl-CoA dehydrogenase (*acads*), short and branched chain specific acyl-CoA dehydrogenase (*acadsb*), and medium-chain specific acyl-CoA dehydrogenase (*acadm*) are the dehydrogenases that catalyze the first step of fatty acid β-oxidation in the mitochondria, and trifunctional enzyme subunit alpha 1 (*hadha1*), trifunctional enzyme subunit alpha 2 (*hadha2*), and enoyl-CoA delta isomerase 1 (*eci1*) catalyze the last step of fatty acid β-oxidation in the mitochondria [25]. Peroxisomal acyl-coenzyme A oxidase 1 and 3 (*acox1* and *acox3*), and acetyl-coenzyme A carboxylase carboxyl transferase subunit alpha 1 (*acca1*) involved in the rate-limiting step of peroxisomal β-oxidation of very long-chain fatty acids [26]. In the present study, high dietary phosphorus escalated the mRNA expression of fatty acid β-oxidation genes (mitochondria: *acad8, acads, acadsb, acadm, hadha1, hadha2, cpt1a, cpt2* and *eci1*; peroxisomes: *acox1, acox3* and *acca1*), suggesting that high dietary phosphorus activated the fatty acid β-oxidation. Taken together, for the first time, our data indicated that high dietary phosphate activated the mitochondrial fatty acid β-oxidation in the intestine.

KLF4 is one of the crucial modulators for fatty acid β-oxidation in the mitochondria [14, 33]. The present study showed that high dietary phosphate and Pi incubation activated the Klf4 mRNA and protein expression, in agreement with other studies [19]. Liao et al. [13] pointed out that KLF4 regulates mitochondrial fatty acid β-oxidation by the ERRα/PGC-1α pathway and PPARα pathway. Zhang et al. [37] found that ERRα/PGC-1α-SIRT3 pathway played important roles in the control of fatty acid β-oxidation in the mitochondria. In the present study, high dietary phosphate and Pi incubation significantly up-regulated the Sirt3 mRNA and protein expression. Thus, it is reasonable to speculate that Sirt3 may be a potential target protein for Klf4. Our further investigation found that *klf4* knockdown attenuated the increase of the Sirt3 protein and mRNA levels induced by Pi and that Klf4 bound to the Errα/Pgc1-α transcriptional module and increased the activity of *sirt3* promoter, which confirmed our speculation. PPARα is an important transcription factor that regulates fatty acid β-oxidation [18]. In our study, high dietary phosphate and Pi incubation significantly increased the Pparα protein and mRNA levels, and *klf4* knockdown attenuated the Pi-induced increase of the Pparα protein and mRNA levels, in agreement with the report in mice [13]. Moreover, for the first time, we predicted the Klf4 response elements (KRE) in the *pparα* promoter and found that KLF4 directly bound to the KRE of *pparα* promoter and mediated Pi-induced changes of the promoter activity, indicating that Pparα is a target protein of Klf4 under dietary phosphorus addition.

LCAD is a rate-limiting enzyme that catalyzes the first step of the mitochondrial fatty acid β-oxidation. Studies pointed out that the elevated deacetylation and transcription level of LCAD contributed to mitochondrial fatty acid oxidation [16, 41]. Chen et al. [16] pointed out that SIRT3 regulated mitochondrial fatty acid β-oxidation via the control of the LCAD acetylation level. Here, Pi incubation significantly increased the Lcad deacetylation levels, and the interaction of Sirt3 and Lcad exists under Pi incubation, in agreement with other report [34]. Thus, our study indicated that Pi incubation reduced the Lcad acetylation level by activating Sirt3, thereby activating the mitochondrial β-oxidation. Other study found that LCAD was one of PPARα target genes [18]. In our study, high levels of dietary phosphate and Pi incubation significantly increased the mRNA and protein expression of Lcad. Mechanistically, for the first time, we found the functional Pparα responsive element (PPRE) in *lcad* promoter. Moreover, Pi incubation activated the transcriptional activity of Lcad by the Pparα binding to the PPRE on the *lcad* promoter. Thus, our data demonstrated that Pparα directly binds to the *lcad* promoter, and mediates Pi-induced increase of *lcad* transcription levels, thereby controlling mitochondrial fatty acid β-oxidation.

## Conclusion

In this study, dietary Pi excess significantly increased lipid degradation in the intestine and IECs. Mechanistically, high phosphate induced lipid degradation via the up-regulation of mitochondrial fatty acid β-oxidation, and Klf4-Sirt3/Pparα-Lcad pathway was required for high phosphorus-induced mitochondrial fatty acid β-oxidation and lipolysis. Mechanistically, Klf4 bound to and cooperated with the Errα/Pgc-1α transcriptional module, and enhanced the promoter activity, transcription and protein levels of *sirt3*, which in turn regulated the deacetylation of Lcad under Pi treatment. Klf4 also regulated the transcriptional level of Lcad which was mediated by Pparα. Thus, our study provided potential preventive targets against excess intestinal lipid deposition in the vertebrates, possibly including human beings.

## Supplementary information

**Additional file 1: Supplemental Text S1.** Yellow catfish primary intestinal epithelial cells (IECs) isolation and culture. **Supplemental Table S1.** Feed formulation and proximate analysis of experimental diets. **Supplemental Table S2.** Primers used for quantitative real-time PCR analysis. **Supplemental Table S3.** Primers used for plasmid construction of expression vector. **Supplemental Table S4.** Primers used for plasmid construction of si-klf4. **Supplemental Table S5.** Primers used for plasmid construction of promoters. **Supplemental Table S6.** Primers used for site-mutation analysis. **Supplemental Table S7.** Primers used for electrophoretic mobility-shift assay. **Supplemental Table S8.** Effects of dietary phosphorus supplementation on growth performance and feed utilization of yellow catfish. **Supplemental Figure S1.** MTT assay of primary IECs under Pi incubation. **Supplemental Figure S2.** Immunofluorescent staining of KLF4 protein under Pi incubation. **Supplemental Figure S3.** Nucleotide sequence of yellow catfish sirt3 promoter. Numbers are relative to the transcription start site (+1). **Supplemental Figure S4.** ERRα response elements (ERE) located at −1443 bp to −1457 bp and KLF4 response elements (KRE) located at −406 bp to −419 bp of pparα promoter. **Supplemental Figure S5.** Relative luciferase activity of sirt3 promoter after the incubation with different overexpression vectors. **Supplemental Figure S6.** The immunoprecipitation experiment for the analysis of KLF4 and ERRα binding with SIRT3. **Supplemental Figure S7.** Nucleotide sequence of yellow catfish pparα promoter. Numbers are relative to the transcription start site (+1). **Supplemental Figure S8.** KLF4 response elements (ERE) located at −1443 bp to −1457 bp (KRE1) and −406 bp to −419 bp (KRE2) of pparα promoter. **Supplemental Figure S9.** Nucleotide sequence of yellow catfish lcad promoter. Numbers are relative to the transcription start site (+1).

## Data Availability

All data generated or analyzed during this study are included in this published article.

## Abbreviations

ATP: adenosine triphosphate
CPT: carnitine palmitoyl transferase
EMSA: electrophoretic mobility shift assay
FAS: fatty acid synthase
H&E: hematoxylin & eosin
Lcad: long chain acyl-CoA dehydrogenase
LD: lipid droplet
Klf4: Krüppel like factor 4
NEFA: non-esterified fatty acid
ORO: Oil red O
Pparα: peroxisome proliferator activated receptor alpha
PVDF: polyvinylidene difluoride
Sirt3: Silent mating-type information regulation 2 homolog 3
TBST: Tris-buffered saline with Tween
TG: triglyceride
