# Supplementary material for: Klf4-Sirt3/Pparα-Lcad pathway contributes to high phosphate-induced lipid degradation
Source: Cell Commun Signal. 2023 Jan 9;21:5. doi: 10.1186/s12964-022-01008-w (PMC9830870; doi:10.1186/s12964-022-01008-w)
Supplement: Supplementary file 2 — Additional file 1: Supplemental Text S1. Yellow catfish primary intestinal epithelial cells (IECs) isolation and culture. Supplemental Table S1. Feed formulation and proximate analysis of experimental diets. Supplemental Table S2. Primers used for quantitative real-time PCR analysis. Supplemental Table S3. Primers used for plasmid construction of expression vector. Supplemental Table S4. Primers used for plasmid construction of si-klf4. Supplemental Table S5. Primers used for plasmid construction of promoters. Supplemental Table S6. Primers used for site-mutation analysis. Supplemental Table S7. Primers used for electrophoretic mobility-shift assay. Supplemental Table S8. Effects of dietary phosphorus supplementation on growth performance and feed utilization of yellow catfish. Supplemental Figure S1. MTT assay of primary IECs under Pi incubation. Supplemental Figure S2. Immunofluorescent staining of KLF4 protein under Pi incubation. Supplemental Figure S3. Nucleotide sequence of yellow catfish sirt3 promoter. Numbers are relative to the transcription start site (+1). Supplemental Figure S4. ERRα response elements (ERE) located at −1443 bp to −1457 bp and KLF4 response elements (KRE) located at −406 bp to −419 bp of pparα promoter. Supplemental Figure S5. Relative luciferase activity of sirt3 promoter after the incubation with different overexpression vectors. Supplemental Figure S6. The immunoprecipitation experiment for the analysis of KLF4 and ERRα binding with SIRT3. Supplemental Figure S7. Nucleotide sequence of yellow catfish pparα promoter. Numbers are relative to the transcription start site (+1). Supplemental Figure S8. KLF4 response elements (ERE) located at −1443 bp to −1457 bp (KRE1) and −406 bp to −419 bp (KRE2) of pparα promoter. Supplemental Figure S9. Nucleotide sequence of yellow catfish lcad promoter. Numbers are relative to the transcription start site (+1). [file 12964_2022_1008_MOESM2_ESM.docx]

Supplemental Text S1

**Yellow catfish primary intestinal epithelial cells (IECs) isolation and culture.**

First, the intestine tissue was prepared for by removing longitudinal muscular layer and washed with ice-cold D-Hank’s Balanced Salt Solution (HBSS) wash solution containing 100U penicillin, 100mg/mL streptomycin, 25mg/mL gentamycin and 0.5 mM dithiothreitol (DTT) (Thermo Fisher Scientific). The tissue was cut into small pieces and washed several times with HBSS wash solution until the supernatant was clear. The contents were allowed to settle for 10 min and the supernatant was discarded.

The remaining tissue was placed in 10 mL of digestion buffer containing 1% v/v fetal bovine serum (FBS), 75 U/mL collagenase XI, 20 μg/ml protease II, and 0.5 mM DTT in DMEM. The tissue was digested in a 28℃ incubator with shaking at 180 rpm for 2 h. A 10-mL dispersion solution (DMEM containing 2% w/v D-sorbitol) was then added to the digestion mixture and the tissue was disassociated by repeatedly pipetting. Remaining tissue debris was discarded and the supernatant containing proliferative crypts was centrifuged at 200g for 8 min. Cell pellets were washed twice in DMEM (high glucose) and resuspended in DMEM containing 10 mM HEPES, 100 U/ml penicillin, 100 mg/ml streptomycin, 10 ng/ml EGF, 1% ITS-A and 2% FBS, and cultured at 28℃ and 5% CO_2_. Media were changed every 3 days. FBS was adjusted to 10% when cells reached confluence after 10–12 days.

Supplemental **Table S1 Feed formulation and proximate analysis of experimental diets.**

| Ingredients (g kg^−1^) | Pi deficiency | Adequate Pi | Pi excess |
| --- | --- | --- | --- |
| Casein | 350 | 350 | 350 |
| Gelatin | 30 | 30 | 30 |
| Fish meal | 50 | 50 | 50 |
| Wheat flour | 250 | 250 | 250 |
| Fish oil | 25 | 25 | 25 |
| Soybean oil | 25 | 25 | 25 |
| Ascorbyl-2-polyphosphate | 10 | 10 | 10 |
| NaCl | 0 | 0 | 0 |
| NaH_2_PO_4_·2H_2_O | 0 | 15 | 30 |
| CaCl_2_ | 15 | 15 | 15 |
| Vitamin premix | 5 | 5 | 5 |
| Mineral premix(P-free) | 5 | 5 | 5 |
| Betaine | 10 | 10 | 10 |
| Yttrium trioxide (Y_2_O_3_) | 0.5 | 0.5 | 0.5 |
| Cellulose | 224.5 | 209.5 | 194.5 |
| Total | 1000 | 1000 | 1000 |
| Proximate analysis (%, dry weight) |  |  |  |
| Moisture | 8.25 | 8.50 | 7.34 |
| Crude protein | 40.85 | 39.22 | 40.51 |
| Crude lipid | 6.54 | 6.60 | 6.50 |
| Crude ash | 2.78 | 3.55 | 4.54 |
| Pi | 0.32 | 0.65 | 0.80 |

Vitamin premix (mg or IU per kg diet): retinylacetate, 10000IU; cholecalciferol, 1000IU; all-rac-a- tocopheryl acetate, 30IU; menadione nicotinamide bisulfite, 7; thiamine hydrochloride, 6; riboflavin, 3; pyridoxine hydrochloride, 12; D-calcium pantothenate, 30; niacin, 50; biotin, 1; folic acid, 6; cyanocobalamine, 0.03.

Mineral mixture (mg per kg diet): NaCl 500, FeSO_4_·7H_2_O, 40; ZnSO_4_·7H_2_O, 40; MnSO_4_·H_2_O, 40; CuSO_4_·5H_2_O, 2; CaIO_3_·6H_2_O, 3; Na_2_SeO_3_, 0.05; CoSO_4_, 0.05.

**Abbreviations:** L-P, low phosphate；I-P, intermediate phosphate; H-P, high phosphate

Supplemental **Table S2 Primers used for quantitative real-time PCR analysis**

| Genes | Forward primer (5’-3’) | Reverse primer (5’-3’) | Accession no. |
| --- | --- | --- | --- |
| *fasn* | AACTAAAGGCTGCTGGTTGCTA | CACCTTCCCGTCACAAACCTC | JN579124 |
| *hsl* | GAAGGACAGGACAATGAGAAGC | TGTACCACCAGCCAAGGAGA | KJ588765 |
| *cpt1a* | ATTTGAAGAAGCACCCAGAGTATGT | CCCTTTTATGGACGGAGACAGA | JQ074177 |
| *srebp1* | CTGGGTCATCGCTTCTTTGTG | TCCTTCGTTGGAGCTTTTGTCT | JX992742 |
| *pparα* | CGAGGATGGGATGCTGGTG | CGTCTGGGTGGTTCGTCTGC | JX992740 |
| *pparγ* | ACGCCCCGTTCGTTATCC | TGAGCAGAGTCACCTGGTCATTG | JX992741 |
| *atgl* | TTGCGGAAATGTGATTGAGGT | CACGGAAGGCAGGAGGGA | KF614123 |
| *klf4* | GGCAACATGTCAGTGTACGG | CACTGACGCTGCATCAAACT | XM027139922 |
| *sirt3* | GTTTGCCAGCTTATCCGGAG | TCCGCGTAGTCATTCCTCTC | XM027153055 |
| *lcad* | CTGGTCATTGTTGTCGCTGT | AATCAGCTGGGAGACGAACA | MG599805.1 |
| *acads* | AAGTAGGCTGCTTTGCTTTGAGTGAG | AATTGGTGATCC  AGGCTTTGGTG | MG599806 |
| *acad8* | ACCATTACATCCTGAACGGCTCCA | GCGAGTTCCAACCAACCTTCTTCT | MG599807 |
| *acadm* | GCAGAAGGAGTTCCAGGAGGTGTC | CAGCAATAATGACCGGCATTTGTC | MG599804 |
| *acadvl* | CGAGACCTGCGAATCTTCCGTAT | AATCTCCCCAGCCAACAAACCAG | MG599809 |
| *acadsb* | ACGGTCGGGAGTTTCTGTTTATCTG | GATGTGAAGCCCCTCAGTGTCGC | MG599810 |
| *hadha1* | GCTTGACGACAGGTTTCGGGTTT | TGTTTAGCTGCTTATCCTTTGATTTGG | MG599815 |
| *hadha2* | CCAGACTTACAACAAGGGGAAGATACA | GGTCATTGATGCGAATAACAGCCAC | MG599814 |
| *hadhb* | CGACATCGACGTGTTCGAGTTCC | GCCATACTGCCCTCCCTCCTTCT | MG599812 |
| *acox1* | GCACCCCTGAGCAGCGTGACAAA | ACCGTCGGGCTGTTCAGAACGAA | MG599802 |
| *acox3* | TGTGACGCCAGACGGACAATACG | TGGTAGAAGCCGCCATTGCTGTA | MG599803 |
| *acaa1* | AGACTCAGTGTTGTTTCCCGTCATTT | TCTGGTGACAGTCCCACATCCTTC | MG599816 |
| *slc34a2* | ACTCACTACAGATCGCTCCG | AGAGACAGACCGAACACCAG | HQ008238.2 |
| *pit1* | CATCCTTGCTACCGTGTTCG | CAAACAGCAGAGCCAACCAT | XM_027175342.1 |
| *pit2* | CATCTGTGGCATGGCTGTAC | TTCCTCCAGCACTTCTCCTG | XM_027167024.1 |
| *xpr1* | GGAGCTGGCCAAAATCAACA | GTCCTTGATGTTGCGGTGTT | XM_027148591.1 |
| *pic* | TCTGGGCTTTCTACAAGGGG | GTTCAGCACAGACACCACAG | XM_027173000.1 |
| *ucp2* | TTGGCCTGTATGACTCGGTT | AATGGTTCGGTAGGCATCCA | XM_027139563.1 |
| *ndufa9* | TTATGCCATACCCCTTGCCA | CGGAGGAAGGAGTGATACCC | XM_027135850.1 |
| *sdha* | GAATCTACCAGCGTGCGTTT | CCCTTCCTCCATCAACAGGT | XM_027163703.1 |
| *sdhb* | AATCAATAACGTACCGCCCG | TTGCACACGAACCGCAGATA | XM_027137463.1 |
| *atpaf1* | TATTCCTGGACTTGTGCCGA | TGTCCAACAGGCTCCTTCTT | XM_027169998.1 |
| *atp5f1a* | GAGAGCTGCCAAGATGAACG | ACACAGACAGGCCCACATTA | XM_027133276.1 |
| *atpaf2* | AGGGTGAAGGTGGTCAGTTT | ATCATTTGGTCCTTGTCGCG | XM_027137658.1 |
| *β-actin* | GGACTCTGGTGATGGTGTGA | CTGTAGCCTCTCTC GGTCAG | EU161066 |
| *rpl7* | GGCAAATGTACAGGAGCGAG | GCCTTGTTGAGCTTGACGAA | KP938522 |
| *hprt* | ATGCTTCTGACCTGGAACGT | TTGCGGTTCAGTGCTTTGAT | KP938523 |
| *tuba* | TCAAAGCTGGAGTTCTCGGT | AATGGCCTCGTTATCCACCA | KP938526 |
| *b2m* | GCTGATCTGCCATGTGAGTG | TGTCTGACACTGCAGCTGTA | KP938520 |
| *ubc9* | TCAAGAAGAGCCAGTGGAGG | TAGGGGTAGTCGATGGGGAA | KP938524 |
| *tbp* | AGCAAAGAGTGAGGAGCAGT | ACTGCTGATGGGTGAGAACA | KP938525 |
| *gapdh* | TTTCAGCGAGAGAGACCCAG | ATGACTCTCTTGGCACCTCC | KP938521 |
| *18s rRNA* | AGCTCGTAGTTGGATCTCGG | CGGGTATTCAGGCGAGTTTG | KP938527 |
| *elfa* | GTCTGGAGATGCTGCCATTG | AGCCTTCTTCTCAACGCTCT | KU886307 |

**Abbreviations:** *fas*, fatty acid synthase; *hsl*, hormone-sensitive lipase; *cpt1a*, carnitine palmitoyl transferase; *srebp1*, sterol regulatory element binding proteins-1; *pparα*, peroxisome proliferator activated receptor alpha; *pparγ*, peroxisome proliferator-activated receptor γ; *atgl*, adipose triglyceride lipase; *klf4*, Krüppel-like factor 4; *sirt3*, sirtuin 3; *lcad*, acyl-CoA dehydrogenase, long chain; *acads*, acyl-CoA dehydrogenase, C-2 to C-3 short chain; *acad8*, acyl-CoA dehydrogenase family member 8; *acadm*, acyl-CoA dehydrogenase medium chain; *acadvl*, acyl-CoA dehydrogenase, very long chain; *acadsb*, acyl-CoA dehydrogenase short/branched chain;, *hadha1,* hydroxyacyl-CoA dehydrogenase trifunctional multienzyme complex subunit alpha 1; *hadha2*, hydroxyacyl-CoA dehydrogenase trifunctional multienzyme complex subunit alpha 2; *hadhb*, hydroxyacyl-CoA dehydrogenase subunit b; *acox1*, acyl-Coenzyme A oxidase 1; *acox3*, acyl-Coenzyme A oxidase 1; *acca1*, acetyl/ propionyl-CoA carboxylase subunit alpha; *slc34a2,* type IIb sodium-dependent phosphate cotransporter*; pit1,* solute carrier family 20 member 1*; pit2,* solute carrier family 20 member 2; *xpr1,* xenotropic and polytropic retrovirus receptor 1; *pic,* solute carrier family 25 member 3; *ucp2,* uncoupling protein 2; *ndufa9,* NADH:ubiquinone oxidoreductase subunit A9a; *sdha,* succinate dehydrogenase complex, subunit A; *sdhb,* succinate dehydrogenase complex, subunit B; *atpaf1,* ATP synthase mitochondrial F1 complex assembly factor 1; *atp5f1a,* ATP synthase F1 subunit alpha; *atpaf2,* ATP synthase mitochondrial F1 complex assembly factor 2; *β-actin*, actin, beta; *rpl7*, ribosomal protein L7; *hprt*, hypoxanthine phosphoribosyl transferase; *tubα*, alpha tubulin; *b2m*, beta-2-microglobulin. *ubc9*, ubiquitin conjugating enzyme 9; *tbp*, TATA-box binding protein; *gapdh*, glyceraldehyde-3-phosphate dehydrogenase; *18S rRNA*, 18S ribosomal RNA; *elfa*, translation elongation factor.

Supplemental **Table S3.** **Primers used for plasmid construction of expression vector**

| Genes | Forward primer (5’-3’) | Reverse primer (5’-3’) |
| --- | --- | --- |
| *klf4* | ctagcgtttaaacttaagcttATGAGGCAGCCTCCAGCAG | aacgggccctctagactcgagGAACTTATCGTCGTCATCCTTGTAATCA |
| *sirt3* | ctagcgtttaaacttaagcttATGCTGAGCAGAATTGGACATG | aacgggccctctagactcgagTTAATGGTGATGGTGATGATGCTT |
| *lcad* | ctagcgtttaaacttaagcttATGATGATGATGATGAAGATGATGATG | aacgggccctctagactcgagTCACTTATCGTCGTCATCCTTGTAA |
| *errα* | ctagcgtttaaacttaagcttATGTCTTCCAGAGAGCGCCG | aacgggccctctagactcgagCTACAGATCCTCTTCAGAGATGAGTTTC |
| *pgc1α* | ctagcgtttaaacttaagcttATGGCGTGGGACAGGTGC | aacgggccctctagactcgagTTAAGCGTAGTCTGGGACGTCGTAT |

Supplemental **Table S4.** **Primers used for plasmid construction of si-*klf4***

| Genes | Forward primer (5’-3’) | Reverse primer (5’-3’) |
| --- | --- | --- |
| si-*klf4*-358 | GCCGAGAUGGAAGGACGAATT | UUCGUCCUUCCAUCUCGGCTT |
| si-*klf4*-797 | GGAUCAGAACCAUCAACUUTT | AAGUUGAUGGUUCUGAUCCTT |
| si-*klf4*-1167 | GGGAAGGCUGUGGAUGGAATT | UUCCAUCCACAGCCUUCCCTT |

Supplemental **Table S5 Primers used for plasmid construction of promoters**

| Gene | Primers | Forward primer (5'-3') | Reverse primer (5'-3') |
| --- | --- | --- | --- |
| *sirt3* | pGl3-1576/+44 | ctatcgataggtaccgagctcCATTCTGGTGGTGGA | cagtaccggaatgccaagcttTATCACTCAGGTCCAACAGA |
| *lcad* | pGl3-1618/+50 | ctatcgataggtaccgagctcCGCAGCATAAATGGC | cagtaccggaatgccaagcttAGCCTGCTGATCGAGTG |
| *pparα* | pGl3-837/+63 | ctatcgataggtaccgagctcCGACGCACTTAATTGGT | cagtaccggaatgccaagcttCCCGAAATAATATCACA |

Supplemental **Table S6 Primers used for site-mutation analysis**

| Gene | Primers | Forward primer (5'-3') | Reverse primer (5'-3') |
| --- | --- | --- | --- |
| *sirt3* | ERE-SIRT3 | agactgatcagtatgacttgATTTTTGGGTCACCTTGTATTTTG | agtcatactgatcagtcttaCAAAATACAAGGTGACCCAAAAAT |
|  | KRE-SIRT3 | aaagcgcatgacatcggacgttTAAGGAAAATATTACATGAGTGAA | tccgatgtcatgcgctttTTAATTTATTCCGATTATCCA |
| *pparα* | KRE1-PPARα | ggatatatacatgagaccaTCTGACAAATGCTGGAAATGTAA | ggtctcatgtatatatccaTTACATTTCCAGCATTTGTCAGA |
|  | KRE2-PPARα | ttagtcgcgtgcggcgaataCCCGTACTGTCTATATTGTCTCTCATC | ttcgccgcacgcgactaataTATGACAGATCAATAATAAAGTGTGAGG |
| *lcad* | PPRE-LCAD | cttcgcatgtgctatgaatgAAATAAACGTGTGCACTGTGTT | ggcactataagtcatctctgtactcAACACAGTGCACACGTTTATTT |

Supplemental **Table S7** **Primers used for electrophoretic mobility-shift assay**

| Primers |  | Forward primer (5'-3') | Reverse primer (5'-3') |
| --- | --- | --- | --- |
| ERE-SIRT3 | Biotin-probe | ATTTTTGGGTCACCTTGTATTTTG | CAAAATACAAGGTGACCCAAAAAT |
|  | Mutative-competitor | ATTTTTGATATGGTCCACATTTTG | CAAAATGTGGACCATATCAAAAAT |
| KRE-SIRT3 | Biotin-probe | TGAGCGCGCGTGCA | TGTGTGCACGCGC |
|  | Mutative-competitor | TGGATATATACATGAGAC | TGGTCTCATGTATATATC |
| KRE1-PPARα | Biotin-Probe | TCTGACAAATGCTGGAAATGTAA | TTACATTTCCAGCATTTGTCAGA |
|  | Mutative-competitor | TCCAGACGCGTACATGCGCTGAA | TTCAGCGCATGTACGCGTCTGGA |
| KRE2-PPARα | Biotin-probe | GTACAGATGCACACTGTCATGC | GCATGACAGTGTGCATCTGTAC |
|  | Mutative-competitor | GTATCACGATCAGTGAGTCTGC | GCAGACTCACTGATCGTGATAC |
| PPRE-LCAD | Biotin-probe | AAATAAACGTGTGCACTGTGTT | AACACAGTGCACACGTTTATTT |
|  | Mutative-competitor | AAAGCGCATGACATCGGACGTT | AACGTCCGATGTCATGCGCTTT |

Supplemental Table S8. Effects of dietary phosphorus supplementation on growth performance and feed utilization of yellow catfish.

|  | LP | IP | HP |
| --- | --- | --- | --- |
| Survival^1^ | 98.89±1.92 | 92.22±8.39 | 86.67±5.77 |
| IBW, g/fish | 2.20±0.04 | 2.20±0.02 | 2.20±0.02 |
| FBW, g/fish | 7.87±0.27^a^ | 10.04±0.11^b^ | 11.49±1.47^b^ |
| WG^2^, % | 257.16±12.76^a^ | 355.47±5.56^b^ | 421.84±66.28^b^ |
| SGR^3^, %/d | 2.27±0.06^a^ | 2.71±0.02^b^ | 2.94±0.22^b^ |
| FCR^4^, g/fish | 1.69±0.09^b^ | 1.47±0.15^b^ | 1.43±0.04^a^ |
| FI^5^, %/d | 5.48±0.42 | 6.00±0.49 | 6.30±0.77 |
| CF^6^, % | 1.94±0.36 | 1.80±0.22 | 1.94±0.30 |

IBW, Initial mean body weight; FBW, final mean body weight; WG, weight gain; SGR, specific growth rate; FI, feed intake. FCR, feed conversion rate; CF, condition factor;

Values are mean ± SEM (n=3 replicate tanks). “a b c” indicate significant difference among the three groups, as determined by one-way ANOVA, and further post hoc Duncan’s multiple range testing. P≤0.05.

^1^Survival= 100 ×final fish number/initial fish number.

^2^WG = (FBW-IBW)/IBW×100;

^3^SGR = 100×[ln (FBW)-ln (IBW)]/d;

^4^FCR = dry food fed (g)/wet weight gain (g);

^5^FI=dry feed fed (g)/fish numbers;

^6^CF = 100×(body weight, g)/(body length, cm)^3^;

Supplemental **Figure S1.** MTT assay of primary IECs under Pi incubation

**
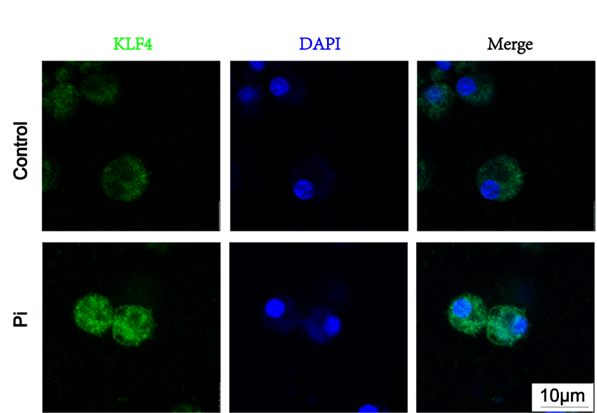
**

Supplemental **Figure S2.** Immunofluorescent staining of KLF4 protein under Pi incubation


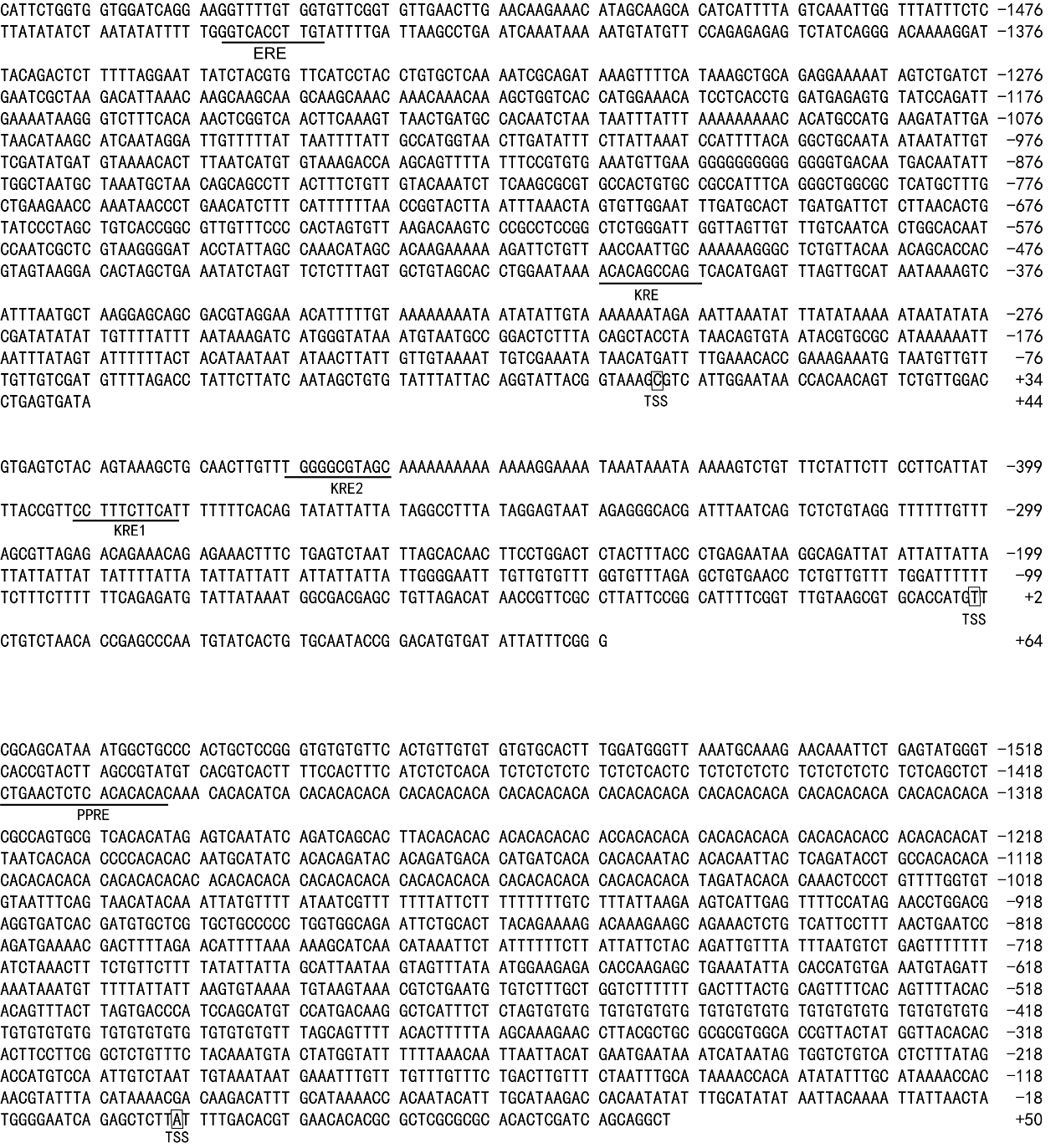


Supplemental **Figure S3.** Nucleotide sequence of yellow catfish *sirt3* promoter. Numbers are relative to the transcription start site (+1).


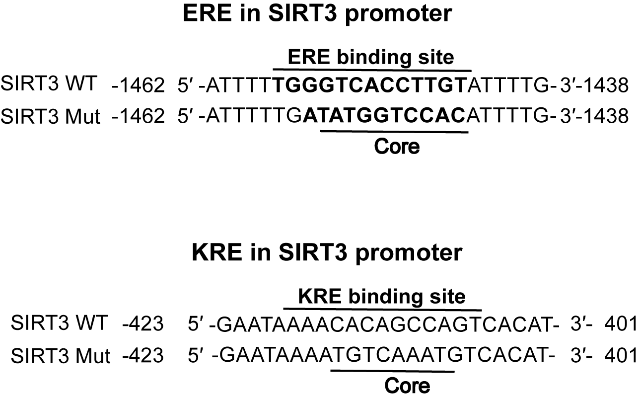


Supplemental **Figure S4.** ERRα response elements (ERE) located at -1443 bp to -1457 bp and KLF4 response elements (KRE) located at -406 bp to -419 bp of *pparα* promoter.


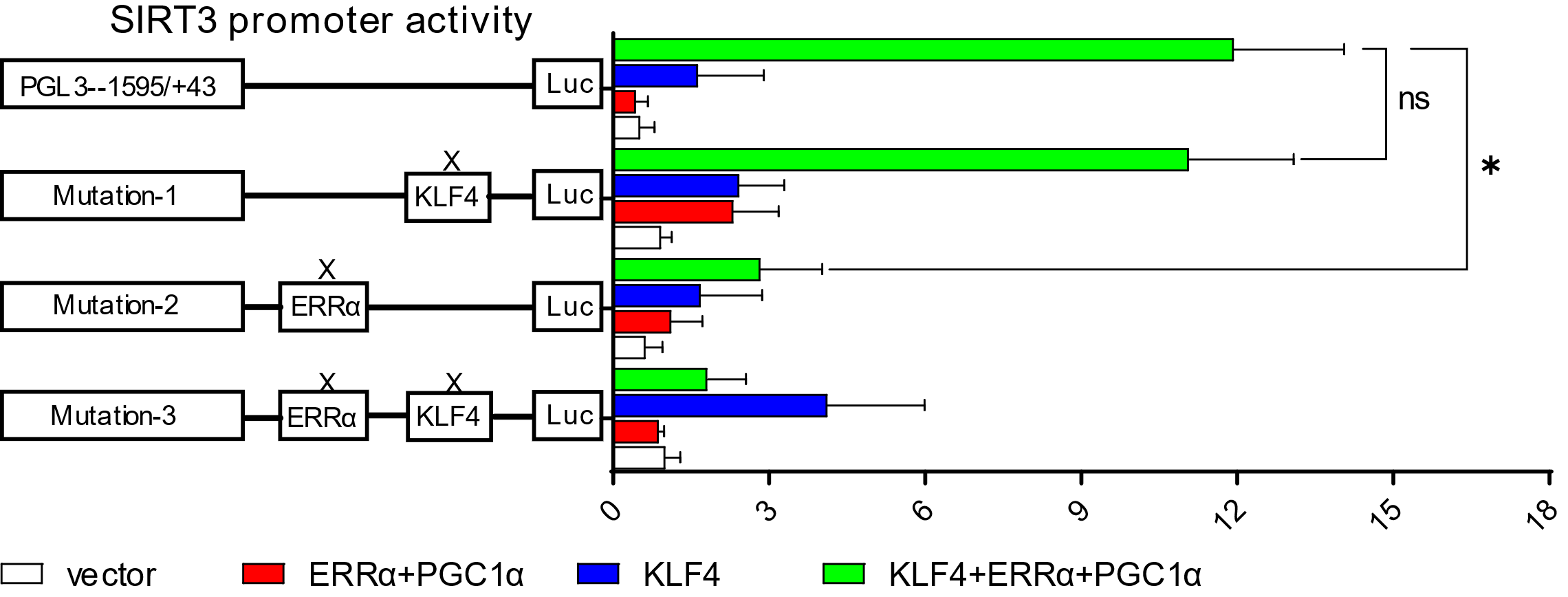


Supplemental **Figure S5.** Relative luciferase activity of *sirt3* promoter after the incubation with different overexpression vectors.


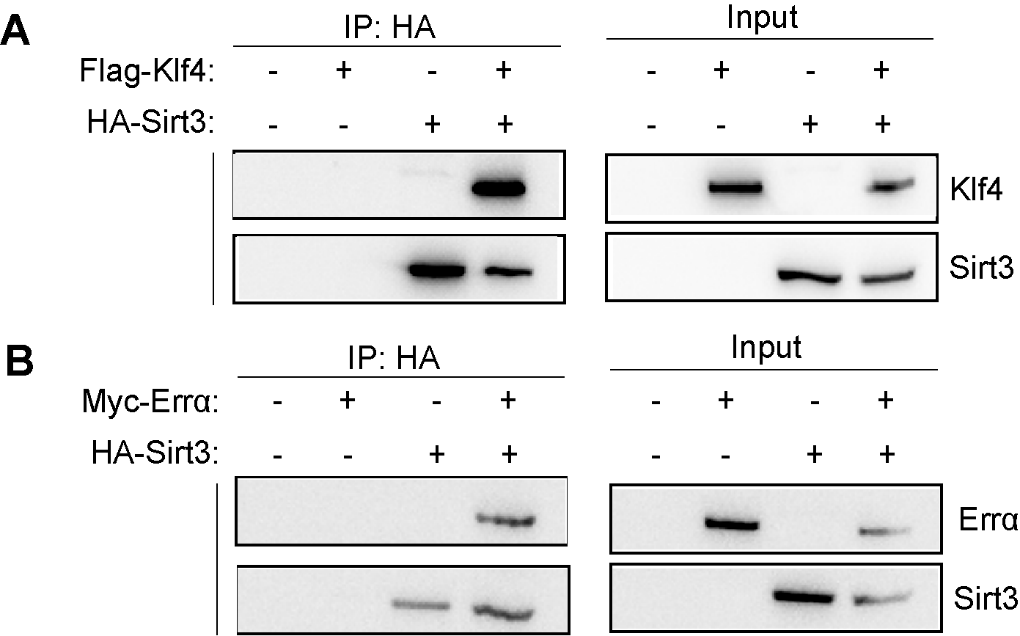


Supplemental **Figure S6. The immunoprecipitation experiment for the analysis of KLF4 and ERRα binding with SIRT3.** (A) Flag-tag Klf4 and HA-tag Sirt3 were transfected into 293T cells, and the interaction between Klf4 and Sirt3 was determined with IP and western blot. (B) Myc-tag Errα and HA-tag Sirt3 were transfected into 293T cells, and the interaction between Errα and Sirt3 was determined with IP and western blot.


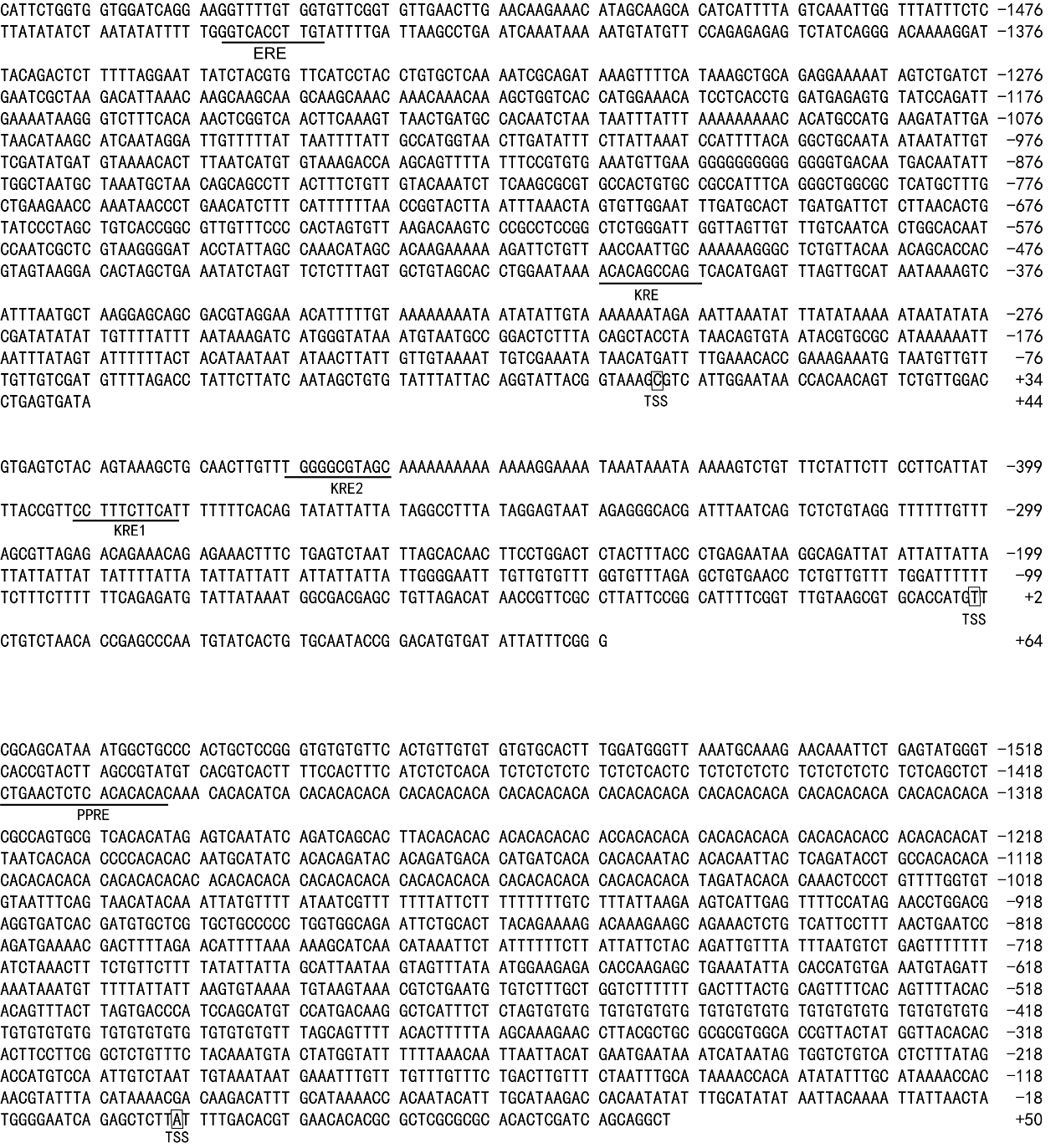


Supplemental **Figure S7.** Nucleotide sequence of yellow catfish *pparα* promoter. Numbers are relative to the transcription start site (+1).

Supplemental **Figure S8.** KLF4 response elements (ERE) located at -1443 bp to -1457 bp (KRE1) and -406 bp to -419 bp (KRE2) of *pparα* promoter.


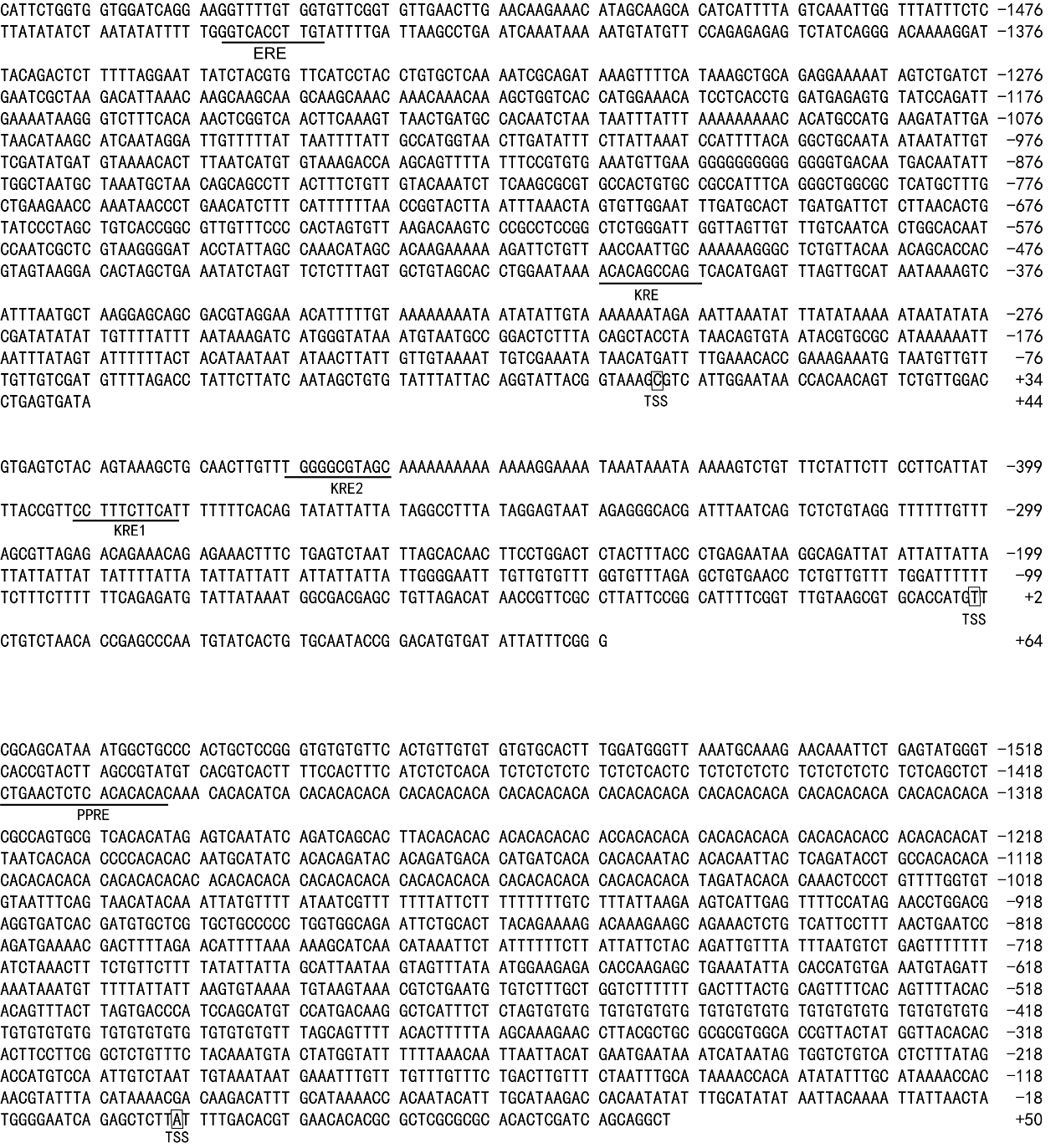


Supplemental **Figure S9.** Nucleotide sequence of yellow catfish *lcad* promoter. Numbers are relative to the transcription start site (+1).
